# Reprogramming of the Antibacterial Drug Vancomycin Results in Potent Antiviral Agents Devoid of Antibacterial Activity

**DOI:** 10.3390/ph13070139

**Published:** 2020-06-29

**Authors:** Zsolt Szűcs, Lieve Naesens, Annelies Stevaert, Eszter Ostorházi, Gyula Batta, Pál Herczegh, Anikó Borbás

**Affiliations:** 1 Department of Pharmaceutical Chemistry, University of Debrecen, Egyetem tér 1, H-4032 Debrecen, Hungary; szucs.zsolt@pharm.unideb.hu (Z.S.); herczegh.pal@pharm.unideb.hu (P.H.); 2 Doctoral School of Pharmaceutical Sciences, University of Debrecen, Egyetem tér 1, H-4032 Debrecen, Hungary; 3 Rega Institute for Medical Research, KU Leuven, B-3000 Leuven; Belgium; lieve.naesens@kuleuven.be (L.N.); annelies.stevaert@kuleuven.be (A.S.); 4 Department of Medical Microbiology, Semmelweis University, Nagyvárad tér 4, H-1089 Budapest, Hungary; ostorhazi.eszter@med.semmelweis-univ.hu; 5 Department of Organic Chemistry, University of Debrecen, H-4032 Debrecen, Hungary; batta@unideb.hu

**Keywords:** glycopeptide antibiotic, vancomycin aglycone hexapeptide, antiviral, influenza virus, human coronavirus

## Abstract

Influenza A and B viruses are a global threat to human health and increasing resistance to the existing antiviral drugs necessitates new concepts to expand the therapeutic options. Glycopeptide derivatives have emerged as a promising new class of antiviral agents. To avoid potential antibiotic resistance, these antiviral glycopeptides are preferably devoid of antibiotic activity. We prepared six vancomycin aglycone hexapeptide derivatives with the aim of obtaining compounds having anti-influenza virus but no antibacterial activity. Two of them exerted strong and selective inhibition of influenza A and B virus replication, while antibacterial activity was successfully eliminated by removing the critical *N*-terminal moiety. In addition, these two molecules offered protection against several other viruses, such as herpes simplex virus, yellow fever virus, Zika virus, and human coronavirus, classifying these glycopeptides as broad antiviral molecules with a favorable therapeutic index.

## 1. Introduction

Seasonal infections by influenza A and B viruses are each year responsible for significant morbidity and mortality [1]. Besides, zoonotic influenza A viruses occasionally enter the human population to cause serious pandemics with a high number of fatalities [2]. Antiviral drugs are essential for influenza treatment and prevention, including in the context of pandemic preparedness. At the moment, four drug classes are available: The M2 ion channel blockers and neuraminidase inhibitors, approved in all countries [3], and two polymerase inhibitors, recently approved in a few countries [4,5]. For each of these drugs, emergence of resistant mutants is possible; this is particularly problematic when the mutant viruses are fit and human-to-human transmissible [6,7,8,9]. Hence, additional drug classes with a distinct mechanism of action remain essential [10,11,12].

Several recent investigations demonstrated that some antibiotics from bacterial origin display interesting antiviral properties [13] representing a unique example of drug repurposing [14]. Glycopeptide antibiotics like teicoplanin, dalbavancin, oritavancin, and telavancin were shown to inhibit Ebola pseudovirus infection [15] and prevent the host cell entry process of Ebola virus, Middle East respiratory syndrome coronavirus (MERS-CoV) and severe acute respiratory syndrome coronavirus (SARS-CoV) [16]. Moreover, semisynthetic hydrophobic derivatives of vancomycin and teicoplanin antibiotics were reported to inhibit HIV [17,18], SARS-CoV [19], HCV [20], and flaviviruses [21]. The anti-influenza virus potential of lipophilic derivatives of glycopeptide antibiotics was first discovered by our group. We reported a class of molecules with various lipophilic modifications at the N-terminal part of the peptide core of ristocetin, showing robust inhibition of influenza virus replication in cell culture [22]. Mechanistic studies with the lead compound demonstrated interference with virus uptake by endocytosis [23]. Encouraged by the favorable selectivity of this compound, we prepared a series of ristocetin and teicoplanin analogues in a systematic manner, to gain further insight in the structure-activity relationship [24,25,26,27,28,29].

Recently, we prepared a series of teicoplanin pseudoaglycone (TC) derivatives by coupling one or two lipophilic side chains to the N-terminus of the glycopeptide core, using triazole, sulfonamide or maleimide linking elements [30]. Some of the modifications yielded remarkably effective inhibitors of influenza A and B viruses with low cytotoxicity. Besides the potent antiviral effect, most analogues proved to be also active against Gram-positive bacteria including vancomycin resistant enterococci. Due to the global threat of antibiotic resistance, the antibacterial activity in this case represents a drawback that could hinder application of these types of derivatives as antiviral agents. The undesirable antibacterial activity of antiviral glycopeptides has been suggested by others as well. Several glycopeptide analogues have been synthesized and evaluated against retroviruses [17,18]. As the antibacterial activity of these derivatives was a concern, the same researchers prepared and evaluated aminodecyl and adamantyl functionalized compounds with partially destroyed peptide cores [31]. These degradation products lacking antibacterial activity typically displayed more-or-less weaker antiviral properties than the intact analogues. Similarly, in the present work we decided to overcome the issue of intrinsic antibacterial activity by using a degraded glycopeptide aglycone, but also exploiting the results of our study on antiviral TC derivatives [30] at the same time.

The first step in the synthetic plan was to select three TC derivatives with optimal antiviral activity combined with low cytotoxicity from our former antiviral study. As for the elimination of the antibacterial activity, obtaining a degradation product with no antibacterial activity from teicoplanin antibiotics is a very difficult task. Even after destroying one or more amide bonds of the peptide core (which are key in binding to the target bacterial cell-wall precursors) some activity still persists [32,33]. The complete elimination of the antibacterial activity requires the concurrent removal of amino acids 1 and 3, which is rather laborious [34]. As such, the simple postliminary degradation of the selected TC derivatives was considered implausible. On the other hand, in the case of vancomycin or its aglycone, the *N*-terminal *N*-methyl-d-leucine moiety can be easily removed by Edman-degradation [35,36] yielding hexapeptide derivatives that are inactive against bacteria, likely due to their inability to bind to the target cell-wall precursors terminating in d-Ala-d-Ala. Since we previously determined, that the antiviral activity of TC derivatives is primarily influenced by the structure of the side chains [30], we envisioned that we could reprogram vancomycin to create selective antiviral agents free of antibacterial activity by incorporating the appropriate lipophilic moieties of the former teicoplanins into vancomycin aglycone hexapeptide.

## 2. Results and Discussion

### 2.1. Chemistry

We synthesized a total of six derivatives by preparing two variants for the three selected side chains. These two variations consist of modifying the vancomycin aglycone hexapeptide in either the *N*- or *C*-terminal position. In this way, we wanted to learn whether the side chain attachment site influences the antiviral activity.

**Scheme 1:**
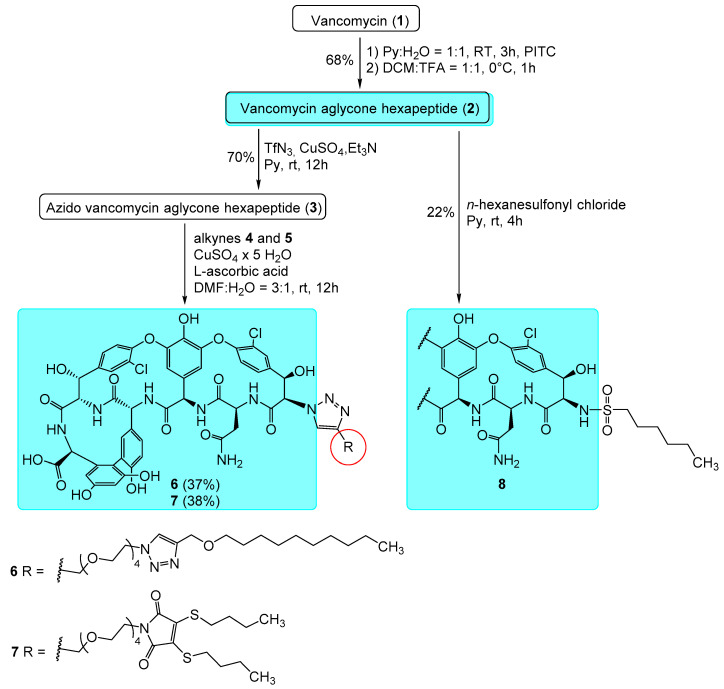
Synthesis and structures of vancomycin aglycone hexapeptide derivatives **6**–**8** modified on the *N*-terminus. See Scheme 2 for the structures of alkynes **4** and **5**.

To synthesize the first variant, we used literature procedures to prepare vancomycin aglycone (**1**) and its hexapeptide derivative (**2**) by the Edman degradation (Scheme 1) [35,36]. Then, by the copper-catalyzed diazotransfer reaction, the *N*-terminal azido derivative (**3**) was successfully prepared in analogy to our previous work [24,30].

**Scheme 2:**
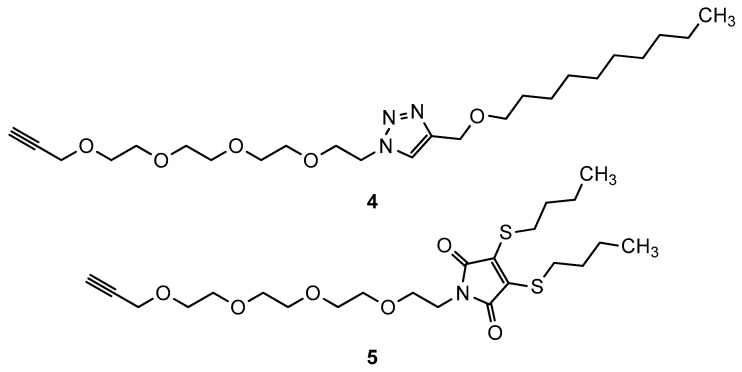
Structures of previously prepared alkynes **4** and **5** used for the synthesis of derivatives **6** and **7**.

The subsequent copper-catalyzed azide-alkyne click reaction using alkyne compound **4** (Scheme 2) yielded triazole derivative **6**. The second compound modified on the *N*-terminus (**7**) was prepared by the same method using the already described maleimide derivative **5 [30]** as the alkyne compound in the final step. The third derivative (**8**) in this group was synthesized using derivative **2** and hexanesulfonyl chloride by sulfonamide formation, similar to what we previously published for teicoplanin congeners [30].

**Scheme 3:**
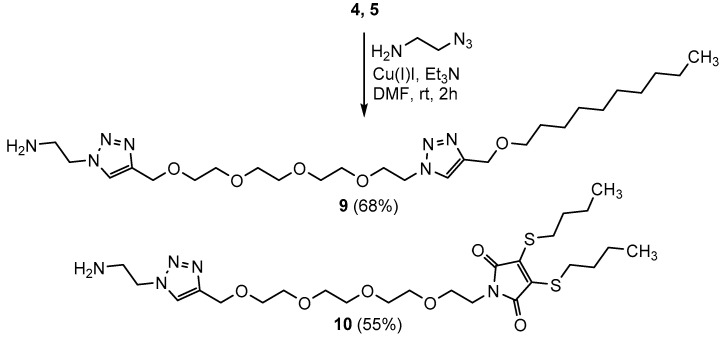
Synthesis of amines **9**-**10** for the *C*-terminal modifications.

As for the *C*-terminal modifications, we decided to prepare two derivatives carrying a 1,2,3-triazole ring, since this moiety often generates bioactive analogues [37]. In order to minimize the structural difference between the side chain in the *N*-versus *C*-terminal position, we used 2-azidoethylamine as a small linker for the synthesis of the appropriate *C*-terminal analogues (Scheme 3).

**Scheme 4:**
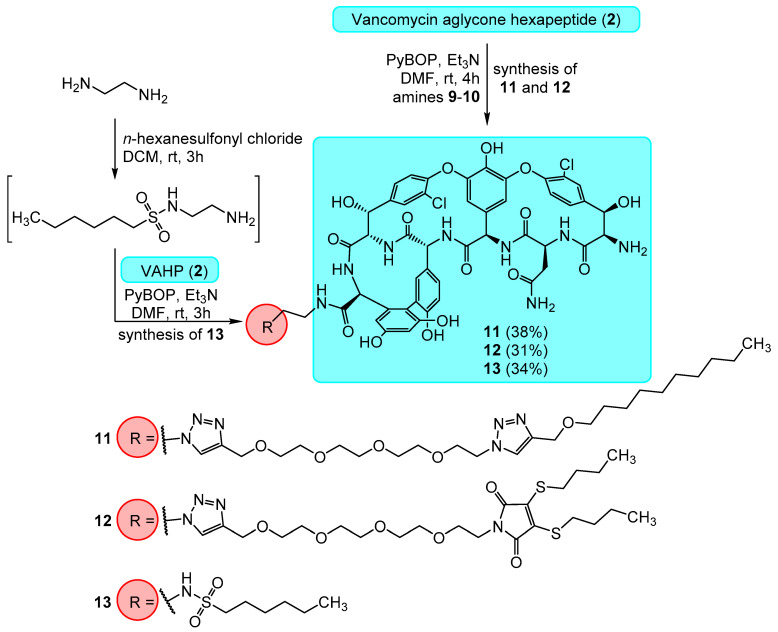
Synthesis and structures of vancomycin aglycone hexapeptide (VAHP) derivatives **11**–**13** modified on the *C*-terminus.

After preparation of the amines, the reaction of compound **2** with amine **9** using the PyBOP reagent gave amide derivative **11** (Scheme 4). Using the same conditions, the reaction between compound **2** and maleimide **10** as the alkyne compound yielded compound **12**. In the case of the *C*-terminal sulfonamide derivative **13** we also used the similar, small linker moiety. In this approach, an amine functionalized sulfonamide was first synthesized from *n*-hexanesulfonyl chloride and ethylenediamine, then the peptide coupling reaction between this compound and compound **2** using PyBOP yielded compound **13**.

### 2.2. Biology

#### 2.2.1. Antibacterial Evaluation

Antibacterial tests were carried out by the broth microdilution method on a panel of eight Gram-positive bacterial strains, using vancomycin and teicoplanin as reference compounds. Neither of the new compounds exhibited significant activity against any bacterium, proving successful elimination of antibacterial activity, as anticipated (Table 1).

#### 2.2.2. Antiviral Evaluation

**Figure 1:**
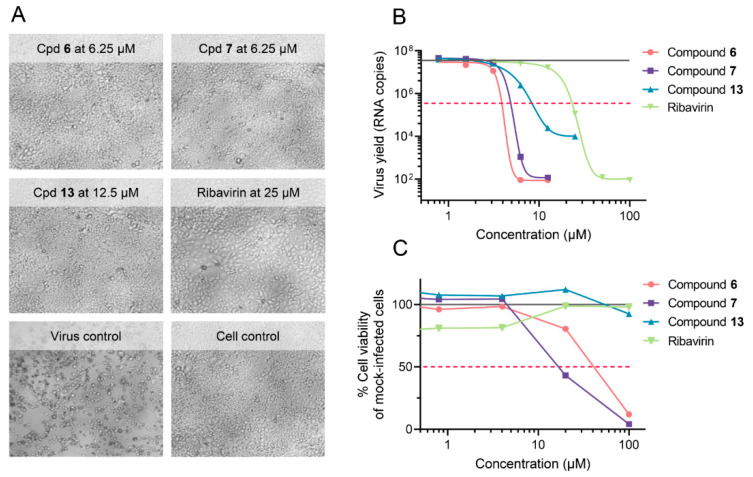
Anti-influenza virus activity of compounds **6**, **7**, and **13**, in MDCK cells at day 3 p.i. with A/PR/8/34 virus. (**A**) Representative images showing complete inhibition of viral cytopathic effect (CPE) at compound concentrations devoid of any cytotoxicity. (**B**) Reduction in virus yield, as determined by RT-qPCR for viral RNA in the supernatant (lower limit of detection: 10^1.6^ copies). Curve fitting by GraphPad Prism, on two data points from one experiment performed in duplicate. Full grey line: Virus yield for untreated virus control; red dashed line: 100-fold reduction in virus yield. (**C**) Compound cytotoxicity in mock-infected cells, determined by MTS cell viability assay (mean data from three experiments). Full grey line: 100% viability in the cell control receiving no compound; red dashed line: 50% cell viability.

With regard to antiviral activity, the two *N*-terminal triazole derivatives **6** and **7** displayed robust activity against the three influenza A or B viruses tested. Upon microscopic inspection, no virus-induced cytopathic effect (CPE) was observed in virus-infected cells treated with 6.25 µM of compound **6** or compound **7** (Figure 1A). The quantitative antiviral efficacy (EC_50_) and cytotoxicity (CC_50_) values, both determined by the MTS cell viability assay, are summarized in Table 2. With EC_50_ values of ~3 µM and a CC_50_ value of 41 µM (compound **6**) and 18 µM (compound **7**), the molecules had a selectivity index (ratio of CC_50_ to EC_50_) of 14 and 6, respectively. Both molecules exhibited clear inhibition of influenza virus replication, since they strongly reduced the virus yield in the supernatant (Figure 1B), giving EC_99_ values of 3.5 µM (compound **6**) and 4.6 µM (compound **7**), which is 5- to 6-fold lower than the EC_99_ for ribavirin (23 µM). At these concentrations, the compounds were devoid of cytotoxicity, as assessed by MTS cell viability assay in mock-infected cells (Figure 1C). The *N*-terminal *n*-hexanesulfonyl derivative **8** and *C*-terminally modified compound **11** proved inactive. For compound **8**, this was somewhat surprising since the analogous teicoplanin pseudoaglycone derivative showed high activity [30]. On the other hand, this result is in line with our previous findings on ristocetin and teicoplanin aglycone derivatives, indicating that even minor structural differences in the peptide core can lead to significantly different anti-influenza virus activity [27]. The *C*-terminally modified compounds **12** and **13** were only slightly active against one or both influenza A virus strains. Compared to compounds **6** and **7**, compound **13** displayed an 8-fold higher antiviral EC_50_ value by MTS assay (Table 2); its lower potency was also evident in the virus yield reduction assay (Figure 1B). This points to the importance of the modification site, since the *C*-terminally modified compounds were clearly inferior to the *N*-terminal analogues.

**Figure 2:**
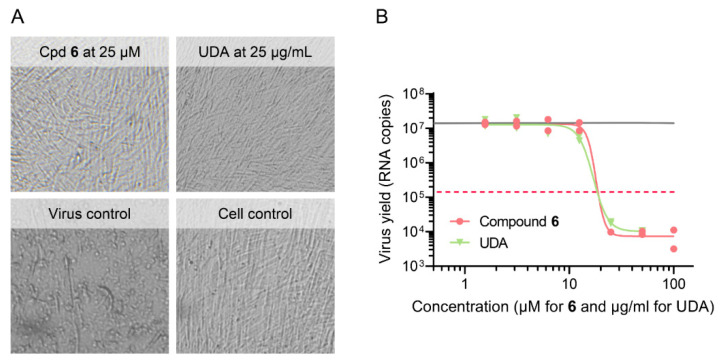
Activity of compound **6** in human embryonic lung (HEL) cells infected with human coronavirus 229E. (**A**) Representative images showing complete inhibition of viral CPE at non-toxic compound concentrations (reference compound: UDA lectin). (**B**) Reduction in virus yield, as determined by RT-qPCR for viral RNA in the supernatant at day 4 p.i. (lower limit of detection: 10^2^ copies). Curve fitting by GraphPad Prism, on two data points from one experiment performed in duplicate. Full grey line: virus yield for untreated virus control; red dashed line: 100-fold reduction in virus yield.

Encouraged by the promising anti-influenza virus activity of compounds **6** and **7**, we tested the two compounds against a range of DNA- and RNA-viruses evaluated in human embryonic lung (HEL) fibroblast, HeLa or Vero cells. For each virus, appropriate reference compounds were included. Protection against virus-induced cytopathicity as well as compound cytotoxicity were determined by the MTS cell viability assay. As shown in Table 3, the two compounds exhibited broad protection against a large variety of viruses, including herpesvirus types 1 and 2 and vaccinia virus. They retained full effectivity against a thymidine kinase deficient form of HSV-1, which was 61-fold (acyclovir) and 89-fold (ganciclovir) resistant to antiherpetic drugs. Moreover, the compounds proved effective against two emerging pathogens for which no therapy is currently approved, i.e., coronavirus (inhibited by compounds **6** and **7**) and Zika virus (inhibited by compound **7**). At a non-toxic concentration of 25 µM of compound **6**, no coronavirus 229E-induced cytopathicity could be observed microscopically (Figure 2A), which agrees with an EC_50_ value of 11 µM as determined by MTS cell viability assay (Table 3). In addition, treatment of infected cells with 25 µM of compound **6** resulted in a 1000-fold reduction of the viral RNA copy number in the supernatant, yielding an EC_99_ value of 20 µM for reduction of virus yield (Figure 2B). Hence, we established, by virus yield assays, that compound **6** suppresses the replication of influenza virus and coronavirus, and for the other viruses, activity was indicated by the protection against viral CPE. This broad activity against distinct viruses fits with our hypothesis that these molecules may act by disrupting the viral endocytosis process, similarly to what we reported for a glycopeptide active against influenza virus [23] and what was described for Ebola virus and MERS and SARS coronaviruses [15,16]. This should become clear from mechanistic work ongoing in our laboratory.

## 3. Materials and Methods

### 3.1. Chemistry

Vancomycin hydrochloride was a gift from TEVA Pharmaceutical Industries Ltd. (Debrecen, Hungary). Vancomycin aglycone hexapeptide, trifluoromethanesulfonyl azide, compounds **4** and **5** were prepared as described elsewhere [24,30]. TLC was performed on Kieselgel 60 F_254_ (Merck) with detection either by immersing into ammonium molybdate-sulfuric acid solution followed by heating or by using Pauly’s reagent for detection. Flash column chromatography was performed using Silica gel 60 (Merck 0.040-0.063 mm) and Silica gel 60 silanized (0.063–0.200 mm). The ^1^H NMR (500MHz, 400 MHz) ^13^C NMR (125 MHz, 100 MHz) and 2D NMR spectra were recorded with a Bruker DRX-400 and Bruker Avance II 500 spectrometers at 300K. Chemical shifts are referenced to Me_4_Si and to the solvent residual signals. MALDI-TOF MS analysis of the compounds was carried out in the positive reflectron mode using a BIFLEX III mass spectrometer (Bruker, Bremen, Germany) equipped with delayed-ion extraction. 2,5-Dihydroxybenzoic acid (DHB) was used as matrix and CF_3_COONa as cationizing agent in DMF. Elemental analysis (C, H, N, S) was performed on an Elementar Vario MicroCube instrument.

#### Synthesis

Synthesis of azido vancomycin aglycone hexapeptide (**3**): 350 mg (0.34 mmol) vancomycin aglycone hexapeptide (**2**) was obtained from 750 mg (0.5 mmol) vancomycin hydrochloride (**1**) by Edman degradation as described in the literature [35,36]. Sodium azide (65 mg, 1.0 mmol) was added to dry pyridine (1.5 mL) cooled to 0–5 °C. Tf_2_O (0.8 mmol, 134 μL) was added dropwise over the course of about 30 min. The reaction mixture was stirred for another 2 h at 0–5 °C. Then, 350 mg (0.34 mmol) of **2** was dissolved in 15 mL pyridine, then 95 μL (2.0 equiv., 0.68 mmol) Et_3_N was added followed by the solution (1.5 mL) of the freshly prepared trifluoromethanesulfonyl azide, and finally 800 μL of a 10 mg/mL CuSO_4_ · 5H_2_O solution. The solution was stirred at room temperature overnight, then the solvents were evaporated. The crude product was dissolved in dilute NH_4_OH, then the pH was set to 1-2 with 1N HCl, the resulting cloudy mixture was extracted with *n*-BuOH three times, the butanolic phase was washed with water, then evaporated and purified by flash column chromatography using step gradient elution (MeCN:H_2_O = 100:0→97:3→94:6→92:8). The title compound was obtained in 250 mg yield (70%) as an off-white powder. NMR: see Table S1 in supporting information. MALDI-MS *m/z* calcd. for C_46_H_37_Cl_2_N_9_O_16_ + Na^+^ [M + Na]^+^: 1064.16. Found: 1064.129.

Synthesis of compound ***6***: 132 mg (0.127 mmol) of **2** was dissolved in 2.0 mL of DMF:H_2_O 3:1 mixture. Next, 72 mg (1.25 equiv., 0.16 mmol) of alkyne **4** was added, followed by 6 mg CuSO_4_ · 5H_2_O. The reaction mixture was stirred at room temperature for 12 h. By this time TLC (cellulose, *n*-PrOH: cc. NH_4_OH = 6:4) indicated good conversion. The solvents were evaporated until a syrupy residue was obtained. Ether was added, and the product was filtered off after precipitation and washed with additional ether to remove the excess alkyne. Purification was carried out by C_18_ reverse phase column chromatography (H_2_O:MeCN = 70:30→60:40→55:45) followed by gel chromatography using Sephadex LH-20 in MeOH. The title compound was obtained as a white powder in 69 mg yield (37%). NMR: see Table S2 in supporting information. Elemental analysis: see Table S8 in supporting information. MALDI-MS *m/z* calcd. for C_70_H_80_Cl_2_N_12_O_21_ + Na^+^ [M + Na]^+^: 1517.48. Found: 1517.68.

Synthesis of compound ***7***: 93 mg (0.09 mmol) of compound **3** was dissolved in 2.0 mL of DMF:H_2_O 3:1 mixture. 54 mg (1.25 equiv., 0.111 mmol) of alkyne **5** was added followed 5 mg CuSO_4_ × 5H_2_O. After 12 h stirring at room temperature, TLC indicated good conversion. The reaction mixture was worked up and purified by C_18_ reverse phase column chromatography as described above. The title compound was obtained as a yellow powder in 52 mg yield (38%). NMR: see Table S3 in supporting information. Elemental analysis: see Table S8 in supporting information. MALDI-MS *m/z* calcd. for C_69_H_74_Cl_2_N_10_O_22_S_2_ + Na^+^ [M + Na]^+^: 1551.369. Found: 1551.367.

Synthesis of compound ***8***: 78 mg (0.077 mmol) of **2** was dissolved in 3 mL dry pyridine and 0.5 mL dry DMF, then 19 μL (1.5 equiv., 0.115 mmol) of *n*-hexanesulfonyl chloride was added. After stirring 3 h at room temperature, ethyl acetate and ether was added, the resulting precipitate was filtered and washed with ether. The crude product was purified by flash column chromatography using Toluene: MeOH = 7:3→6:4 as eluent, followed by gel chromatography using Sephadex LH-20 with MeOH: H_2_O = 6:4 as eluent. The yield was 20 mg (22%). NMR: see Table S4 in supporting information. Elemental analysis: see Table S8 in supporting information. MALDI-MS *m/z* calcd. for C_52_H_51_Cl_2_N_7_O_18_S + Na^+^ [M + Na]^+^: 1186.228. Found: 1186.276.

Synthesis of 2-(4-(13-(4-((decyloxy)methyl)-1H-1,2,3-triazol-1-yl)-2,5,8,11-tetraoxatridecyl)-1H-1,2,3-triazol-1-yl)ethanamine (**9**): 207 mg of **4** [24,30] (0.46 mmol) and 39 mg (0.46 mmol) of 2-azidoethylamine were dissolved in 2 mL dry DMF under argon. 70 μL (1.08 equiv., 0.5 mmol) Et_3_N was added, then 17 mg (20 mol%, 0.09 mmol) Cu(I)I. The reaction mixture was stirred at room temperature for an hour. After evaporation of the solvents, the crude product was purified by flash column chromatography using DCM: MeOH = 95:5 (+0.1% *v/v* NH_4_OH) as eluent. The title compound was obtained as an off white solid in 68% yield (171 mg).

^1^H NMR (400 MHz, CDCl_3_) δ 7.73 (s, 2H, 2 x triazole C*H*), 4.69 (s, 2H, NC*H*_2_), 4.61 (s, 2H, NC*H*_2_), 4.53 (t, *J* = 5.1 Hz, 2H), 4.41 (s, 2H), 3.87 (t, *J* = 5.0 Hz, 2H), 3.76–3.55 (m, 14H, 7 × C*H*_2_), 3.51 (t, *J* = 6.7 Hz, 2H), 1.59 (p, *J* = 6.9 Hz, 2H), 1.35–1.21 (m, 14H, 7 × C*H*_2_), 0.88 (t, *J* = 6.8 Hz, 3H). ^13^C NMR (101 MHz, CDCl_3_) δ 123.71 (2C, 2 x triazole *C*H), 70.95, 70.62, 70.56, 69.86, 69.58, 64.81, 64.35 (10C 10 × C*H*_2_), 50.30 (N–*C*H_2_), 31.97, 29.76, 29.67, 29.65, 29.57, 29.39, 26.21, 22.75 (8C, 8 × *C*H_2_), 14.20 (*C*H_3_). MALDI-MS *m/z* calcd. for C_26_H_49_N_7_O_5_ + Na [M + Na]^+^: 562.37. Found: 562.37.

Synthesis of 1-(1-(1-(2-aminoethyl)-1H-1,2,3-triazol-4-yl)-2,5,8,11-tetraoxatridecan-13-yl)-3,4-bis(butylthio)-1H-pyrrole-2,5-dione (**10**): 208 mg (0.43 mmol) of compound **5 [30]** and 44 mg (1.2 equiv., 0.52 mmol) of azidoethylamine were dissolved in 2 mL dry DMF under argon. 66 μL (1.1 equiv., 0.47 mmol) Et_3_N was added followed by 16 mg (20 mol%) Cu(I)I. The reaction mixture was stirred for 2 h at room temperature. After evaporation the crude product was purified by flash column chromatography using DCM: MeOH = 100:0→93:7 (+0.1% v/v NH_4_OH) as eluent. The yield was 154 mg (55%), yellow syrup.

^1^H NMR (400 MHz, CDCl_3_) δ 7.77 (s, 1H), 4.69 (s, 2H, OC*H*_2_), 4.44 (t, *J* = 5.9 Hz, 2H, N–C*H*_2_), 3.73–3.57 (m, 16H, 8 x C*H*_2_), 3.28 (t, *J* = 7.4 Hz, 4H, 2 x SC*H*_2_), 3.21 (t, *J* = 5.9 Hz, 2H), 1.99 (s, 2H, N*H*_2_), 1.63 (p, *J* = 7.4 Hz, 4H), 1.45 (h, *J* = 7.5 Hz, 4H), 0.93 (t, *J* = 7.3 Hz, 6H). ^13^C NMR (101 MHz, CDCl_3_) δ 166.42 (2C, 2 × *C*=O), 144.81 (triazole *C*_q_), 135.61 (2C, 2 × S-*C*_q_), 123.27 (triazole *C*H), 70.30, 69.80, 69.54, 67.75, 64.47 (8C, 8 x O*C*H_2_), 53.01 (N-*C*H_2_), 41.79, 37.64 (2 x *C*H_2_), 32.32, 31.36, 21.48 (6C, 6 × *C*H_2_), 13.43 (2C, 2 × *C*H_3_). MALDI-MS *m/z* calcd. for C_25_H_43_N_5_O_6_S_2_ + Na^+^ [M + Na]^+^: 596.25. Found: 596.238.

Synthesis of compound ***11**:* Vancomycin aglycone hexapeptide **2** (90 mg, 0.089 mmol) was dissolved in 1.0 mL dry DMF. 95 mg (2.0 equiv., 0.177 mmol) of compound **9** was added followed by 25 μL (2.0 equiv., 0.177 mmol) Et_3_N and 55 mg (1.2 equiv., 0.107 mmol) of PyBOP. The reaction mixture was stirred for 2 h at room temperature, after which TLC (*n*-PrOH:NH_4_OH = 7:3, cellulose) indicated complete conversion. The product was precipitated by the addition of 100 mL of cold EtOAc:Et_2_O = 1:1 mixture, filtered off and washed thoroughly with diethyl ether. The crude product was purified by gel column chromatography using Sephadex LH-20 in MeCN:H_2_O = 8:2 mixture, followed by flash column chromatography in MeCN:H_2_O = 9:1 mixture. The product was obtained as a white powder in 52 mg yield (38%). NMR: see Table S5 in supporting information. Elemental analysis: see Table S8 in supporting information. MALDI-MS *m/z* calcd. for C_72_H_86_Cl_2_N_14_O_20_ + Na^+^ [M + Na]^+^: 1559.54. Found: 1559.76.

Synthesis of compound ***12**:* Vancomycin aglycone hexapeptide **2** (90 mg, 0.089 mmol) was dissolved in 1.0 mL dry DMF, then 101 mg (2.0 equiv., 0.177 mmol) of compound **10** was added followed by 25 μL (2.0 equiv., 0.177 mmol) of Et_3_N and 55 mg (1.2 equiv., 0.107 mmol) of PyBOP. The reaction was stirred for 2 h at room temperature, after which TLC (*n*-PrOH:NH_4_OH = 7:3, cellulose) indicated complete conversion. The product was worked up and purified as described for compound **12**. The title compound was obtained as a yellow powder in 44 mg yield (31%). NMR: see supporting information. NMR: see Table S6 in supporting information. Elemental analysis: see Table S8 in supporting information. MALDI-MS *m/z* calcd. for C_71_H_80_Cl_2_N_12_O_21_S_2_ + Na^+^ [M + Na]^+^: 1593.43. Found: 1593.44.

Synthesis of compound ***13**:* Step 1: 837 μL (12.5 mmol) ethylenediamine was dissolved in 5 mL dry DCM and stirred at room temperature, while 115 mg (0.63 mmol) *n*-hexanesulfonyl chloride in 0.5 mL dry DCM was added via syringe over the course of about 30 min. The reaction mixture was stirred for 4 h, after which it was thoroughly evaporated. The crude product was purified by flash column chromatography using hexanes: acetone = 6:4 (+0.2% *v/v* Et_3_N) as eluent. 95 mg of *N*-(2-aminoethyl)hexane-1-sulfonamide was obtained as a slightly yellow syrup with acceptable purity (based on ^1^H and ^13^C NMR spectra), which was suitable for the amide coupling in Step 2: Vancomycin aglycone hexapeptide **2** (90 mg, 0.089 mmol) was dissolved in 1.0 mL of dry DMF:DMSO mixture. 25 μL Et_3_N (~2 equiv., 0.18 mmol) was added followed by 70 mg *N*-(2-aminoethyl)hexane-1-sulfonamide (~4 equiv., 0.34 mmol) obtained in step 1, followed by 54 mg (1.15 equiv., 0.103 mmol) PyBOP. The reaction was stirred at room temperature for 3 h, then the product was precipitated by the addition of ethyl acetate, filtered out and washed with diethyl ether. The product was purified by flash column chromatography using step gradient elution (toluene: MeOH = 7:3→1:1) then by gel column chromatography (Sephadex LH-20, acetone:H_2_O = 1:1). The title compound was obtained as an off-white powder in 36 mg yield (34%). NMR: see Table S7 in supporting information. Elemental analysis: see Table S8 in supporting information. MALDI-MS *m/z* calcd. for C_54_H_57_Cl_2_N_9_O_17_S + Na^+^ [M + Na]^+^: 1228.29. Found: 1228.27.

### 3.2. Antiviral Procedures

#### 3.2.1. Anti-Influenza Virus Activity

For antiviral testing, 25 mM compound stocks were prepared in 100% DMSO and stored at 4 °C. The compounds were fully soluble under these conditions. In the antiviral tests, the highest concentration tested was 100 µM, corresponding to a non-toxic DMSO content of 0.4%.

The virus strains (A/H1N1: A/Ned/378/05 and A/PR/8/34; A/H3N2: A/Victoria/361/11; and B/Ned/537/05) were propagated in embryonated hen eggs. The antiviral procedure was published elsewhere [38,39]. Madin-Darby canine kidney (MDCK) cells were seeded at 7500 cells per well into 96-well plates, using infection medium (UltraMDCK medium (Lonza) with 225 mg/L sodium bicarbonate, 2 mM L-glutamine, and 2 µg/mL *N*-tosyl-L-phenylalanine chloromethyl ketone (TPCK)-treated trypsin). One day later, virus (MOI: 0.001) was added together with 1:5 serial compound dilutions, to reach a total volume of 200 µL per well. Besides the test compounds, two references were included, i.e., zanamivir and ribavirin (positive controls) plus a condition receiving medium instead of compound (negative control). In parallel, the compound dilutions were also added to a mock-infected plate (in which medium was added instead of virus), to determine compound cytotoxicity. Each plate contained two wells in which all reagents yet no cells were added, to serve as blanks in the MTS calculations. After three days incubation at 35 °C, viral CPE was first monitored by microscopy. Then, the supernatants were replaced by MTS reagent (CellTiter 96^®^ AQ_ueous_ MTS Reagent from Promega) diluted 1:10 in PBS, and 4 h later, absorbance at 490 nm was measured in a plate reader. From all OD values, the blank OD was subtracted. The % protection against virus was defined as: [(OD_Cpd_)virus−(OD_Contr_)virus)]/[(OD_Contr_)mock−(OD_Contr_)virus] × 100, where (OD_Cpd_)virus is the OD for a given concentration of the compound in virus-infected cells; (OD_Contr_)virus is the OD for the untreated virus control; and (OD_Contr_)mock is the OD for the untreated mock-infected control. The % cytotoxicity was defined as: [1−(OD_Cpd_)mock/[(OD_Contr_)mock] × 100, where (OD_Cpd_)mock is the OD for a given concentration of the compound in mock-infected wells. The values for EC_50_ (50% antivirally effective concentration) and CC_50_ (50% cytotoxic concentration) were calculated by interpolation based on semi-log dose response.

To monitor the inhibitory effect of the compounds on virus replication, MDCK cells were seeded, infected (with A/PR/8/34 virus; MOI: 0.001) and treated with 1:2 serial compound dilutions. The plate contained three virus controls (receiving no compound) and two cell controls (receiving no virus and no compound). At day 3 p.i., supernatants were collected and frozen at –80 °C, to quantify the virus yield by one-step qRT-PCR analysis of viral copy number [40]. Two µl supernatant was mixed with 10 μL resuspension buffer and 1 µl lysis reagent (CellsDirect One-Step RT-qPCR kit; Invitrogen) and heated during 10 min at 75 °C. Next, 10 μL lysate was transferred to a qPCR plate containing the qRT-PCR enzymes and buffer (CellsDirect One-Step RT-qPCR kit; Invitrogen), and influenza virus M1-specific primers and probe [40]. The program consisted of: 15 min at 50 °C; 2 min at 95 °C; and 45 cycles of 15 s at 95 °C followed by 90 s at 60 °C. Absolute quantification of vRNA copies was performed by including an M1-plasmid standard. The EC_99_ values were calculated by interpolation and defined as the compound concentration causing 100-fold reduction in vRNA copy number, as compared to the virus control receiving no compound. It was ascertained that the cell controls showed no detectable qPCR signal.

#### 3.2.2. Other Antiviral Procedures

The viruses were propagated and evaluated in the following cell lines: Human embryonic lung (HEL) fibroblast cells, used for human coronavirus 229E [41], herpes simplex virus type 1 (HSV-1 strain KOS, including a thymidine kinase deficient HSV-1/TK^-^ mutant), herpes simplex virus type 2 (HSV-2, strain G) and vaccinia virus (strain Lederle); human cervixcarcinoma HeLa cells, used for respiratory syncytial virus (RSV, strain Long), and African Green Monkey kidney Vero cells, used for yellow fever virus (vaccine strain 17D) and Zika virus (strain MR766). The medium used for virus infection was Dulbecco’s Modified Eagle’s Medium supplemented with 2% fetal calf serum. To prepare virus stocks, confluent cell cultures in 75-cm^2^ flasks were infected with the virus and frozen after 3 to 5 days incubation at 37 °C (or 35 °C in case of human coronavirus 229E), when full-blown CPE was visible. After freeze-thawing and centrifugation, the clarified lysates were stored at −80 °C. For the antiviral experiments, the cells were grown in 96-well plates until confluent. Virus was added (MOI: 0.001) together with 1:5 serial dilutions of the compounds. For each virus, appropriate reference compounds were included. The compound dilutions were also added to a mock-infected plate, to determine compound cytotoxicity. When manifest CPE was reached, i.e., after 3 to 5 days incubation at 37 °C (or 35 °C in case of human coronavirus 229E), CPE and compound cytotoxicity were quantified by the MTS assay, and EC_50_ and CC_50_ values were calculated as explained above for influenza virus.

To assess inhibition of human coronavirus 229E replication, HEL cells were infected and treated with 1:2 serial compound dilutions. The plate contained three virus controls (receiving no compound) and two cell controls (receiving no virus and no compound). At day 4 p.i., supernatants were frozen at −80 °C to determine the virus yield by one-step RT-qPCR assay. Two microliters supernatant was mixed with 11 µl lysis mix containing lysis enhancer and resuspension buffer at a 1:10 ratio (CellsDirect One-Step RT-qPCR kit; Invitrogen), and heated for 10 min at 75 °C. Five microliters of lysate was transferred to a PCR plate containing 9.75 µl RT-qPCR mix (CellsDirect One-Step RT-qPCR) and 0.25 µl Superscript III RT/Platinum Taq enzyme, and coronavirus-229E N-gene specific primers and probe (forward primer 5′-TTAGAGAGCGTGTTGAAGGTG-3′; reverse primer 5′-GTTCTGAATTCTTGCGCCTAAC-3′; probe 5′-FAM-TCTGGGTTG-ZEN-CTGTTGATGGTGCTA-IBFQ-3′). The RT-qPCR program consisted of 15 min at 50 °C, 2 min at 95 °C, and 50 cycles of 15 s at 95 °C and 45 s at 60 °C. An N-gene plasmid standard was included for absolute quantification. Compound activity was expressed as the EC_99_ value, i.e., the concentration causing 100-fold reduction in vRNA copy number, as compared to the virus control receiving no compound. It was ascertained that the cell controls showed no detectable qPCR signal.

## 4. Conclusions

Starting from vancomycin, we have successfully prepared two derivatives with strong activity against influenza virus. The modifications that we introduced were based on our previous work on the glycopeptide antibiotic teicoplanin. Interestingly, some of these modifications yielded compounds **6** and **7** having the same antiviral potency as the analogous teicoplanin pseudoaglycon derivatives [24,30] but lacking antibacterial activity. The short work described here validates that the glycopeptide scaffold is an underexplored entity to conceive new antivirals with a broad activity spectrum, that besides influenza virus includes emerging pathogens like coronavirus and Zika virus.

## Figures and Tables

**Table 1:** Antibacterial evaluation of the new vancomycin aglycone hexapeptide derivatives.

Bacteria	In Vitro MIC in μg/mL							
	TEI	VAN	6	7	8	11	12	13
*B. subtilis* ATCC 6633	0.5	0.5	32	32	32	32	256	256
*S. aureus* MSSA ATCC 29213	0.5	0.5	128	256	256	256	256	256
*S. aureus* MRSA ATCC 33591	0.5	0.5	128	256	256	256	256	256
*S. epidermidis* ATCC 35984	4	2	32	32	32	256	128	128
*S. epidermidis* mecA	16	4	32	32	64	256	256	128
*E. faecalis* ATCC 29212	1	1	32	32	32	128	128	64
*E. faecalis* 15,376 VanA	256	256	128	256	256	256	256	256
*E. faecalis* ATCC 51,299 VanB	0.5	128	128	256	128	256	256	128

TEI: teicoplanin, VAN: vancomycin.

**Table 2:** Anti-influenza virus activity and cytotoxicity in MDCK ^1^ cells.

Compound	CC_50_^2^ (µM)	Antiviral EC_50_^3^ (μM)		
		Influenza A/H1N1	Influenza A/H3N2	Influenza B
**6**	41	4.1	1.4	3.2
**7**	18	3.6	2.0	3.2
**8**	100	>100	>100	>100
**11**	≥20	>100	>100	>100
**12**	100	12	>100	>100
**13**	>100	34	14	>100
Ribavirin	>100	7.0	6.4	7.2
Zanamivir	>100	0.4	9.0	4.5

^1^ Madin Darby canine kidney cells. Virus strains: A/H1N1: A/Ned/378/05; A/H3N2: A/Victoria/361/11; and B/Ned/537/05. ^2^ 50% Cytotoxic concentration based on the formazan-based MTS cell viability assay. ^3^ 50% Effective concentration, i.e., concentration producing 50% inhibition of virus-induced cytopathic effect, as determined by the MTS cell viability assay.

**Table 3:** Evaluation of compounds **6** and **7** against a broad range of DNA- and RNA-viruses ^1^.

Compound	CC_50_^2^ (µM)			Antiviral EC_50_^3^ (µM) - cell line ^4^							
	HEL	HeLa	Vero	HEL					HeLa	Vero	
				HSV-1	HSV-2	HSV-1/TK^-^	Vaccinia Virus	Human Coronavirus 229E	RSV	Yellow Fever Virus	**Zika Virus**
**6**	>100	>100	>100	20	7.8	7.4	7.2	11	7.7	>100	>100
**7**	>100	>100	>100	43	6.5	11	32	32	60	55	14
Cidofovir	>250	>250	>250	2.4	1.0	5.8	37	-	-	-	-
Aciclovir	>250	>250	>250	2.4	0.05	146	>250	-	-	-	-
Ganciclovir	>100	>100	>100	0.1	0.03	8.9	>100	-	-	-	-
UDA ^5^	>100	>100	>100	-	-	-	-	1.8	-	-	-
Ribavirin	>250	>250	>250	-	-	-	-	-	5.0	119	-
Mycophenolic acid	>100	>100	>100	-	-	-	-	-	-	0.7	0.8

^1^ Viruses: herpes simplex virus type 1 (HSV-1) or type 2 (HSV-2); a thymidine-kinase deficient (TK^-^) mutant of HSV-1; vaccinia virus; human coronavirus 229E; respiratory syncytial virus (RSV); yellow fever virus and Zika virus. ^2^ 50% Cytotoxic concentration, assessed in mock-infected cells by the MTS cell viability assay. ^3^ 50% Effective concentration offering 50% protection against virus-induced CPE, as determined by the MTS assay. ^4^ Cell lines: HEL, human embryonic lung fibroblast cells; HeLa, human cervix carcinoma cells; Vero, African Green monkey kidney cells. ^5^ UDA: Urtica dioica agglutinin lectin; for this compound, concentrations are expressed in µg/mL.

## Supplementary Materials

The following are available online at https://www.mdpi.com/1424-8247/13/7/139/s1, Table S1: NMR data for Compound **3**, Table S2: NMR data for Compound **6**, Table S3: NMR data for Compound **7**, Table S4: NMR data for Compound **8**, Table S5: NMR data for Compound **11**, Table S6: NMR data for Compound **12**, Table S7: NMR data for Compound **13**, Table S8: Elemental analysis data (C, H, N, S) for vancomycin derivatives **6**–**8**, **11**–**13**.
