# Supplementary material for: Reprogramming of the Antibacterial Drug Vancomycin Results in Potent Antiviral Agents Devoid of Antibacterial Activity
Source: Pharmaceuticals (Basel). 2020 Jun 29;13(7):139. doi: 10.3390/ph13070139 (PMC7407158; doi:10.3390/ph13070139)
Supplement: Supplementary file 1 [file pharmaceuticals-13-00139-s001.pdf]

SUPPLEMENTARY DATA

Reprogramming of the antibacterial drug vancomycin results in potent antiviral agents devoid of antibacterial activity

Zsolt Szűcs[a,b], Lieve Naesens[c], Eszter Ostorházi[d], Gyula Batta[e], Pál Herczegh,[a] Anikó Borbás[a]

[a]Department of Pharmaceutical Chemistry, University of Debrecen, Egyetem tér 1, H-4032 Debrecen, Hungary

[b]University of Debrecen, Doctoral School of Pharmaceutical Sciences

[c]Rega Institute for Medical Research, KU Leuven, B-3000 Leuven,

[d]Department of Medical Microbiology, Semmelweis University, Nagyvárad tér 4, H-1089 Budapest, Hungary

[e]Department of Organic Chemistry, University of Debrecen, H-4032 Debrecen, Hungary

Table of Contents

NMR data and NMR spectra for Compounds **3**, **6-13** (**Tables S1-S7**)...................................... S2

Elemental analysis data for glycopeptide derivatives (**Table S8**) S26

Compound 3

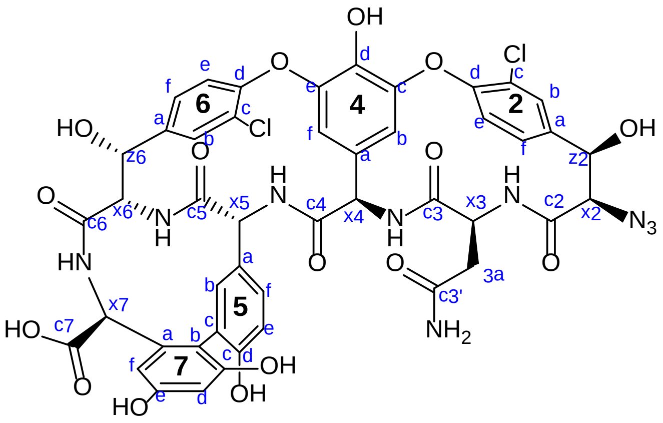

Table S1. NMR data for Compound 3					
Assignment	^{1}H	^{13}C	Assignment	^{1}H	^{13}C
c7	-	174.7	6c	-	125.8
c3'	-	169.9	2f	7.63	128.3
c6	-	167.2	2e	7.20	123.6
c2	-	165.0	6e	7.23	123.1
7e	-	157.5	5c	-	122.9
7c	-	156.0	7b	-	117.6
5d	-	155.3	5e	6.70	116.0
4e	-	147.4	7f	6.58	107.6
4c	-	147.6	4b	5.43	106.8
2d	-	151.3	4f	5.30	104.7
6d	-	149.1	7d	6.31	101.6
6a	-	nd.			
2a	-	138.0	z2	5.29	73.0
7a	-	139.3	z6	5.18	71.0
5b	7.26	136.2	3a,a'	2.34;2.14	38.4
4d	-	134.3			
4a	-	129.2	x6	4.20	61.5
2c	-	126.6	x2	4.60	68.7
2b	7.16	128.8	x7	4.32	59.0
5a	-	126.2	x4	5.67	54.1
6b	7.86	127.5	x5	4.50	53.4
5f	6.73	124.7	x3	4.75	51.0
6f	7.46	127.0			

nd. = not detected

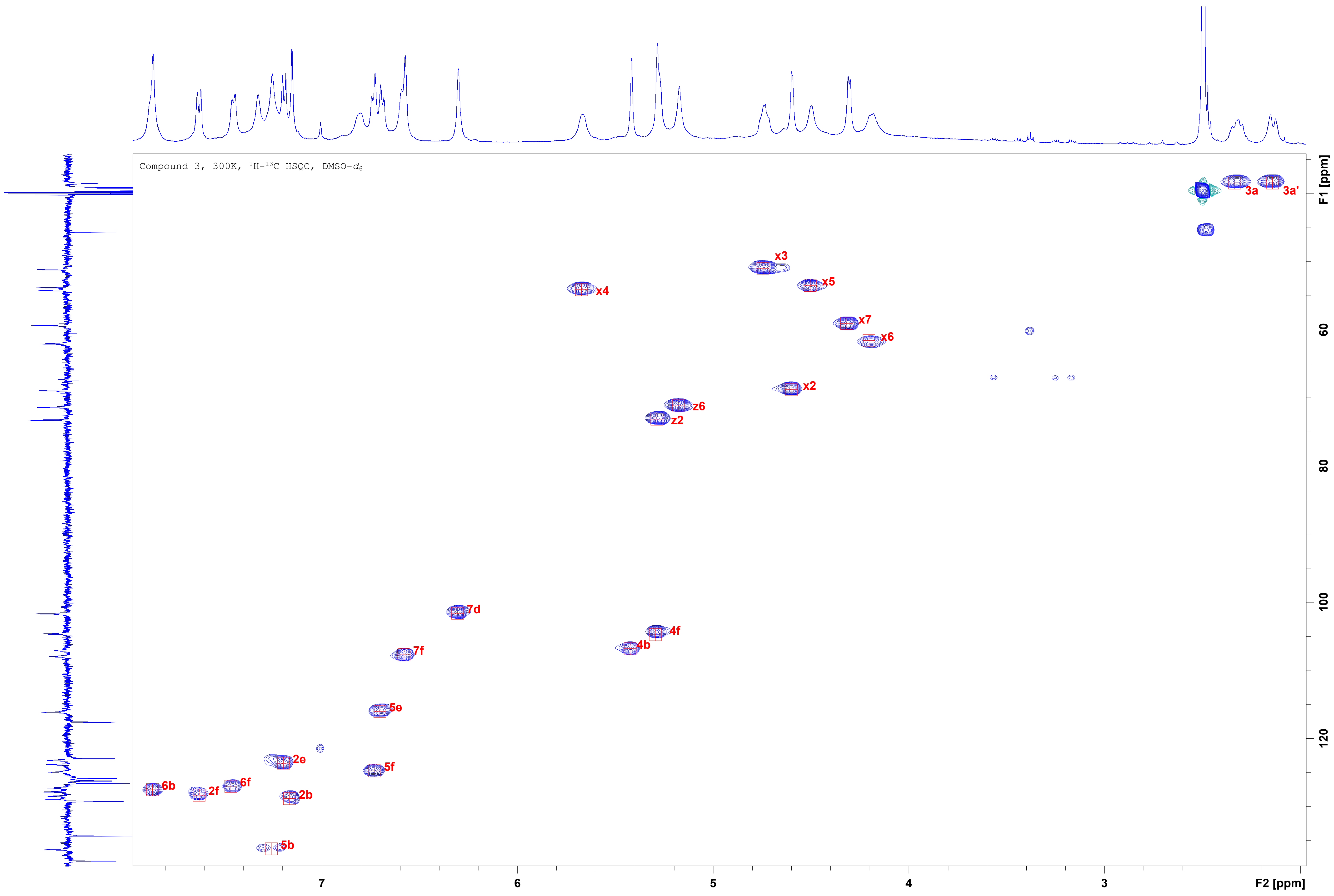

Compound 3, 300K, ^{1}H-^{13}C HSQC, DMSO-d_6
3a
3a'
x3
x5
x4
x7
x6
x2
z6
z2
7d
4f
4b
7f
5e
2e
5f
6b
2f
6f
2b
5b
F1 [ppm]
60
80
100
120
7
6
5
4
3
F2 [ppm]

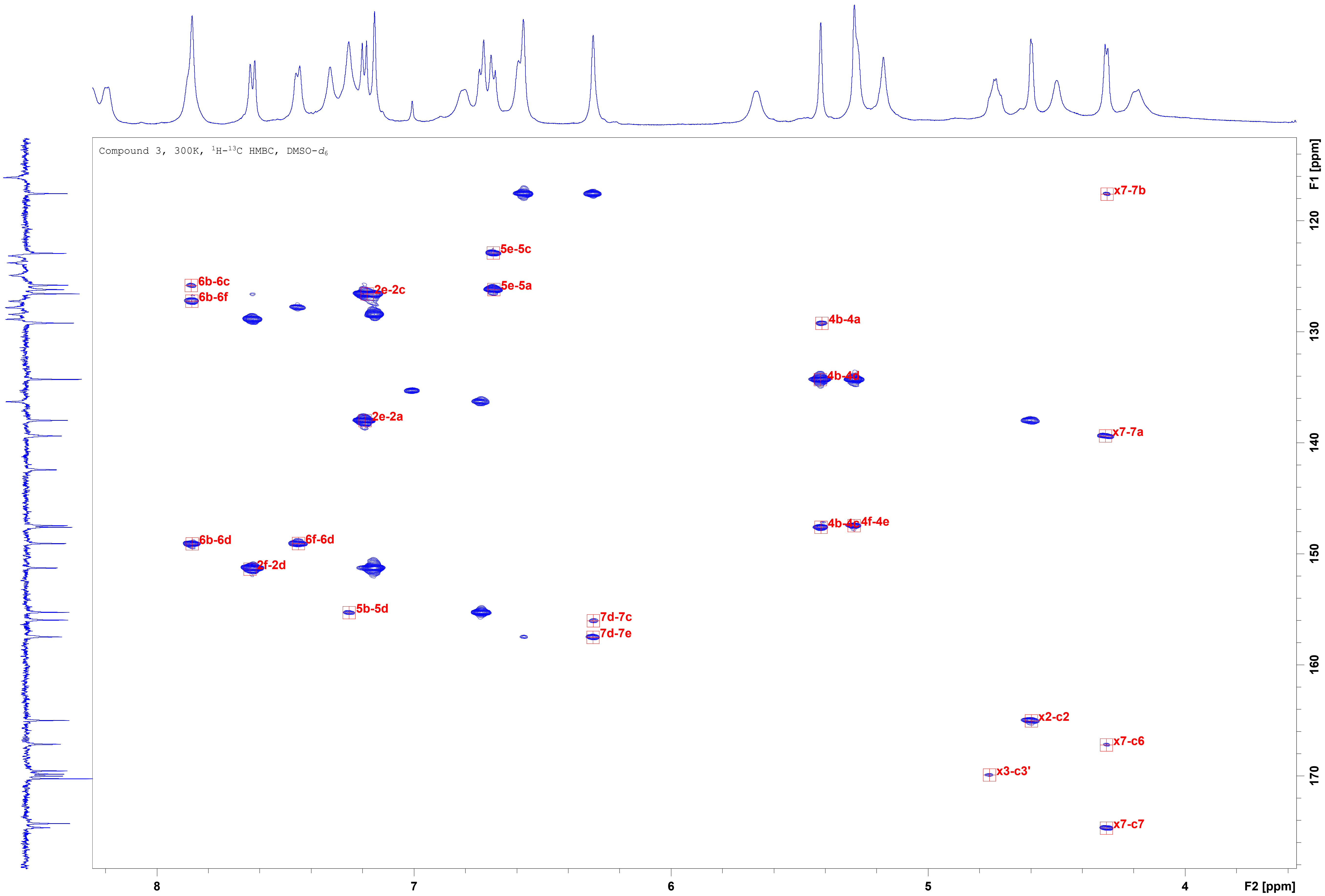

Compound 3, 300K, ^{1}H-^{13}C HMBC, DMSO-d_6
x7-7b
5e-5c
6b-6c
6b-6f
2e-2c
5e-5a
4b-4a
4b-4d
2e-2a
x7-7a
4b-4e
4f-4e
6b-6d
6f-6d
2f-2d
5b-5d
7d-7c
7d-7e
x2-c2
x7-c6
x3-c3'
x7-c7
F1 [ppm]
120
130
140
150
160
170
8
7
6
5
4
F2 [ppm]

Compound 6

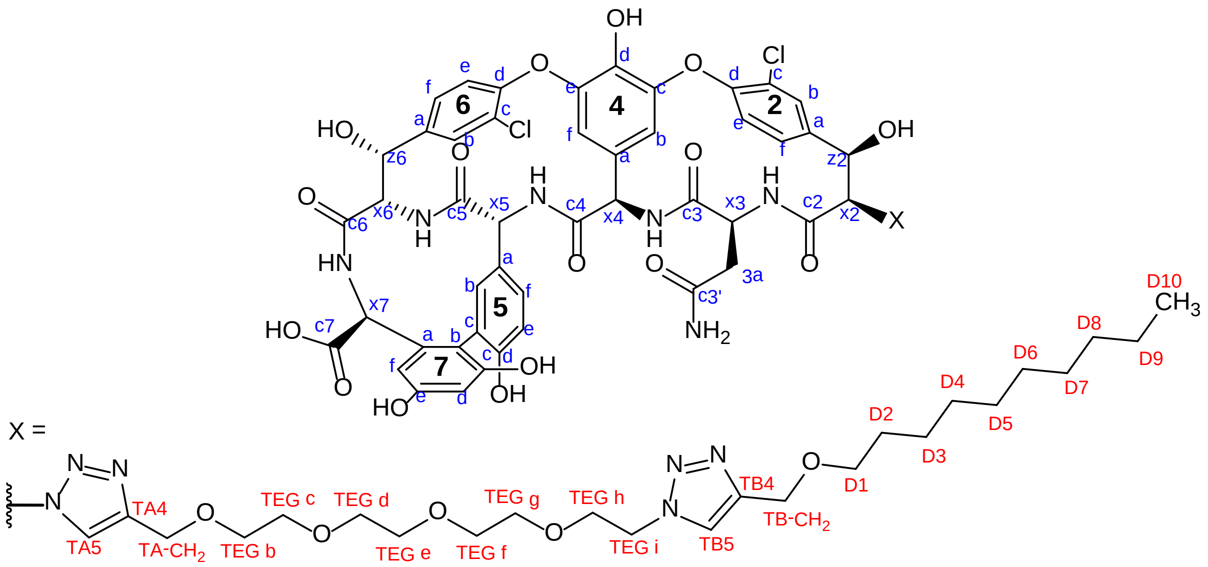

Table S2. NMR data for Compound 6

Assignment	^{1}H	^{13}C	Assignment	^{1}H	^{13}C	Assignment	^{1}H	^{13}C
c7	-	174.6	6f	7.46	127.4	TA-CH_2	4.59	63.4
c4	-	168.7	6c	-	126.2	TA4	-	143.1
c2	-	163.6	2f	7.77	129.2	TA5	8.40	125.7
c6	-	167.0	2e	7.29	124.3	TB-CH_2	4.47	63.3
7e	-	157.4	6e	7.23	123.1	TB4	-	144.3
7c	-	155.9	5c	-	122.6	TB5	8.04	124.1
5d	-	155.1	7b	-	117.6			
4e	-	147.3	5e	6.67	116.0	TEG b	3.59	68.9
4c	-	148.0	7f	6.62	108.1	TEG CH_2	3.50	69.7
2d	-	150.2	4b	5.72	107.3	TEG h	3.81	68.6
6d	-	148.7	4f	5.19	104.5	TEG i	4.50	49.2
6a	-	142.6	7d	6.29	101.7			
2a	-	139.2	z6	5.19	71.2	D1	3.41	69.5
7a	-	139.5	z2	5.28	72.5	D2	1.48	28.8
5b	7.21	136.3				D3	1.25	25.3
4d	-	133.8	x6	4.17	61.8	D4-7	1.29-1.21	29.1
4a	-	129.3	x2	6.10	67.0			29.0
2c	-	126.6	x7	4.32	59.3			28.9
2b	7.51	127.5	x4	5.79	54.7			28.7
5a	-	125.8	x5	4.50	53.5	D8	1.24	31.0
6b	7.89	127.7	x3	4.11	52.0	D9	1.26	22.0
5f	6.74	124.7	3a,a’	2.93;2.17	34.5	D10	0.86	13.8

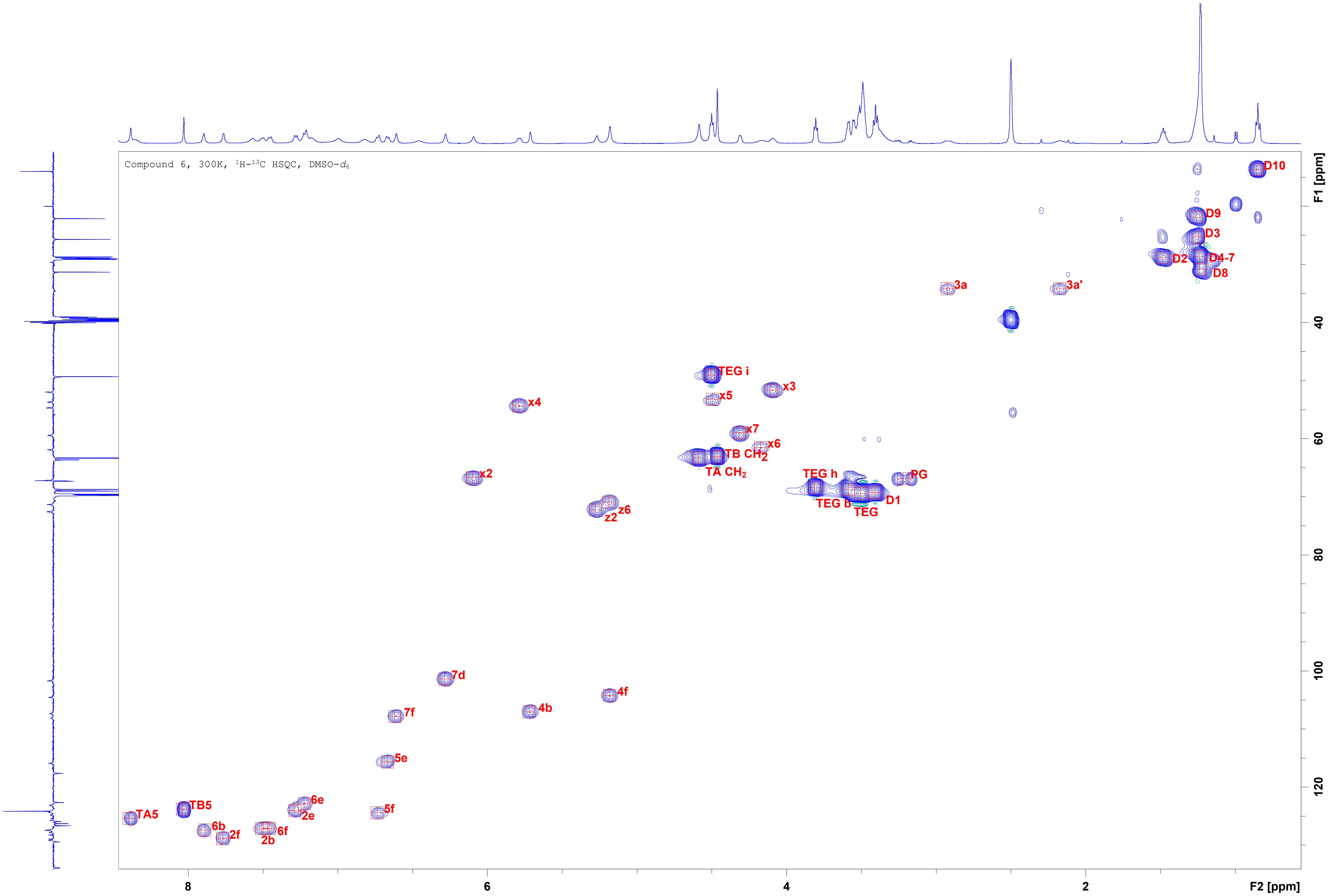

Compound 6, 300K, ^1H-^{13}C HSQC, DMSO-d_6
D10
D9
D3
D2
D4-7
D8
3a
3a'
TEG i
x3
x5
x4
x7
x6
TB CH_2
TA CH_2
x2
TEG h
PG
D1
TEG b
TEG
z6
z2
7d
4f
4b
7f
5e
TB5
6e
TA5
2e
5f
6b
2f
6f
2b
F1 [ppm]
F2 [ppm]
8
6
4
2
40
60
80
100
120

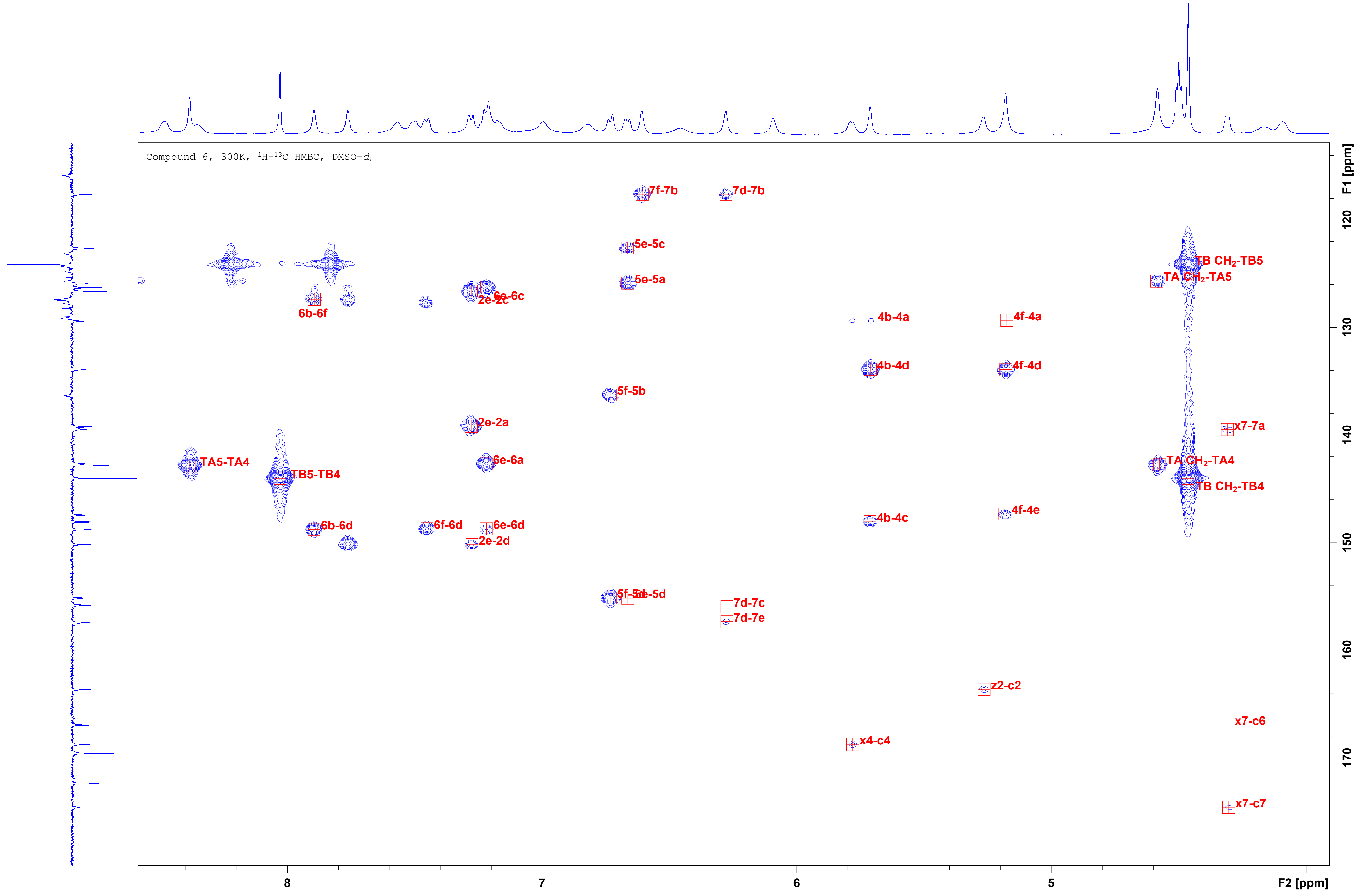
Compound 6, 300K, 1H-13C HMBC, DMSO-d6
7f-7b
7d-7b
5e-5c
5e-5a
TB CH2-TB5
TA CH2-TA5
6b-6f
2e-2c
6e-6c
4b-4a
4f-4a
4b-4d
4f-4d
5f-5b
2e-2a
x7-7a
6e-6a
TA5-TA4
TB5-TB4
TA CH2-TA4
TB CH2-TB4
4f-4e
4b-4c
6b-6d
6f-6d
6e-6d
2e-2d
5f-5d
5e-5d
7d-7c
7d-7e
z2-c2
x7-c6
x4-c4
x7-c7
F1 [ppm]
120
130
140
150
160
170
8
7
6
5
F2 [ppm]

Compound 7

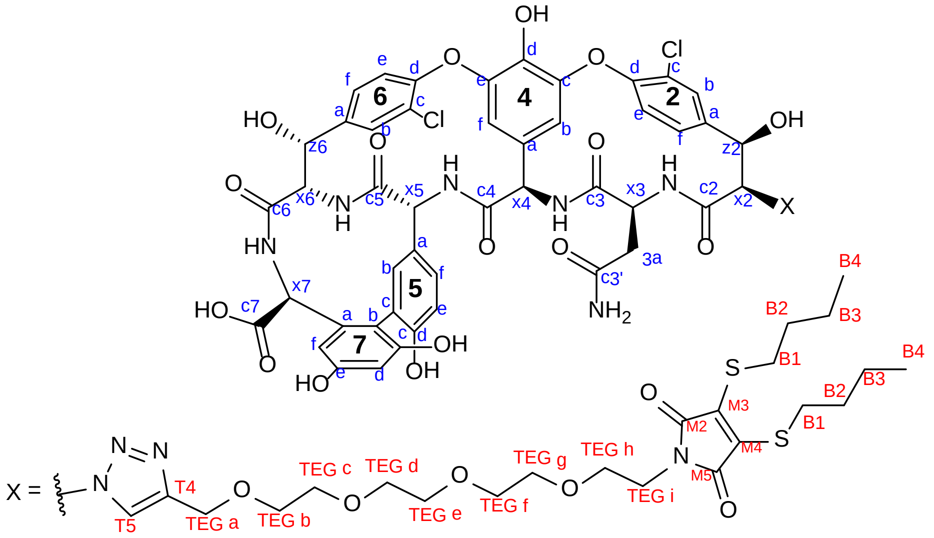

Table S3. NMR data for Compound 7								
Assignment	^{1}H	^{13}C	Assignment	^{1}H	^{13}C	Assignment	^{1}H	^{13}C
c7	-	174.9	5f	6.74	124.5	x5	4.49	53.5
c4	-	168.9	6f	7.45	127.1	x3	4.09	51.7
c6	-	166.9	6c	-	126.3			
7e	-	157.4	2f	7.77	128.8	3a,a’	2.91;2.16	34.4
7c	-	155.8	2e	7.28	124.0			
5d	-	155.2	6e	7.23	122.8	T5	8.38	125.4
4e	-	147.5	5c	-	122.6	T4	-	142.8
4c	-	148.1	7b	-	117.7	TEG b	3.59	68.8
2d	-	150.2	5e	6.67	115.6	TEG CH_2	3.49	69.5
6d	-	148.8	7f	6.62	107.8	TEG h	3.52	66.4
6a	-	142.7	4b	5.71	107.2	TEG i	3.57	37.5
2a	-	139.3	4f	5.17	104.4	T-CH_2	4.59	63.5
7a	-	139.4	7d	6.29	101.4			
5b	7.20	135.9	z6	5.18	71.2	M2,5	-	166.1
4d	-	133.9	z2	5.27	72.3	M3,4	-	135.5
4a	-	129.4						
2c	-	126.6	x6	4.17	61.7	B1	3.25	30.5
2b	7.50	127.1	x2	6.09	67.1	B2	1.55	31.9
5a	-	125.9	x7	4.33	59.1	B3	1.37	20.7
6b	7.90	127.5	x4	5.79	54.5	B4	0.87	13.2

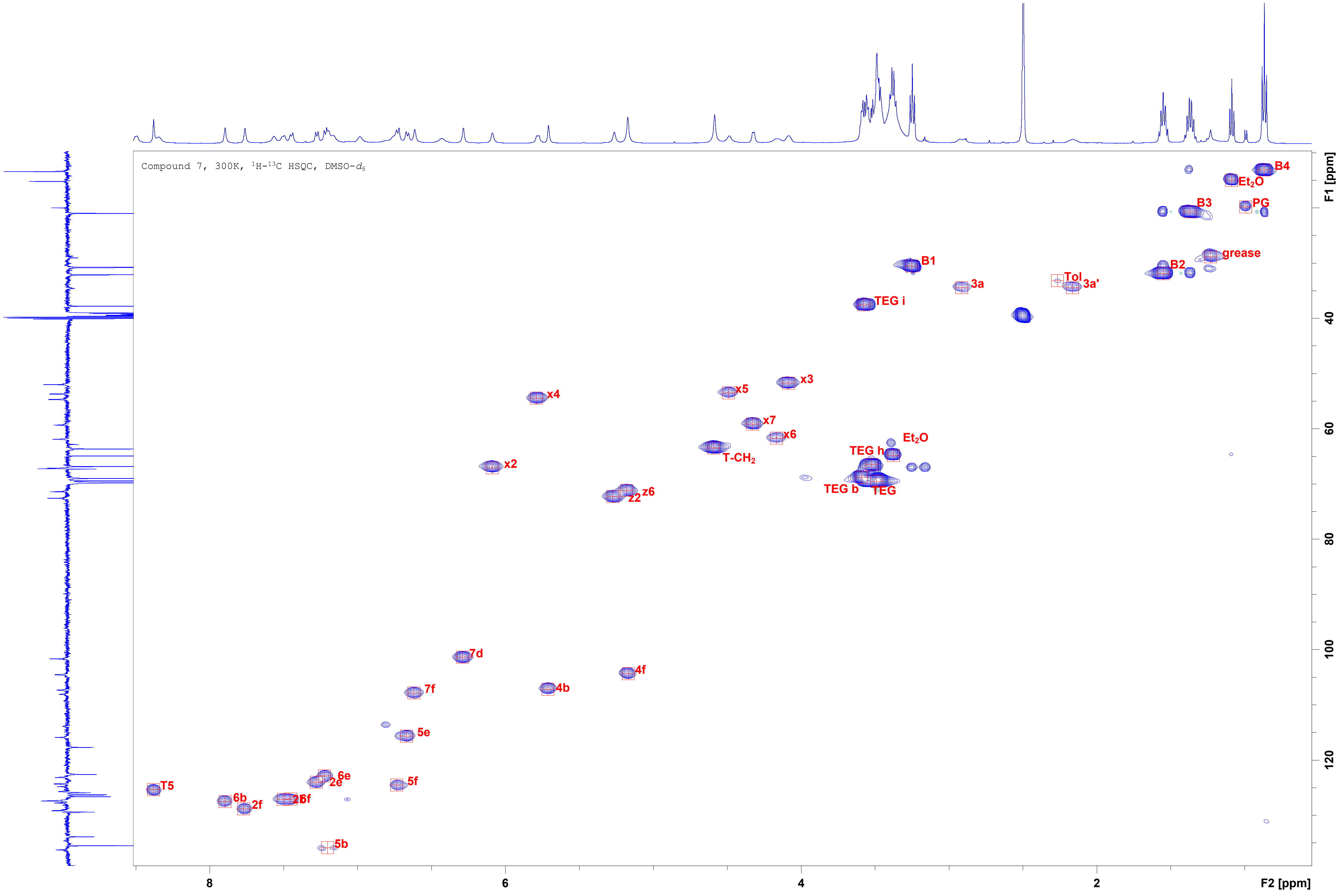

Compound 7, 300K, $^{1}H-^{13}C$ HSQC, DMSO-d_6
B4
Et_2O
B3
PG
grease
B1
B2
3a
Tol
3a'
TEG i
x4
x5
x3
x7
x6
Et_2O
TEG h
T-CH_2
x2
z2
z6
TEG b
TEG
7d
4f
7f
4b
5e
6e
2e
T5
6b
2f
2b
6f
5f
5b
F1 [ppm]
F2 [ppm]

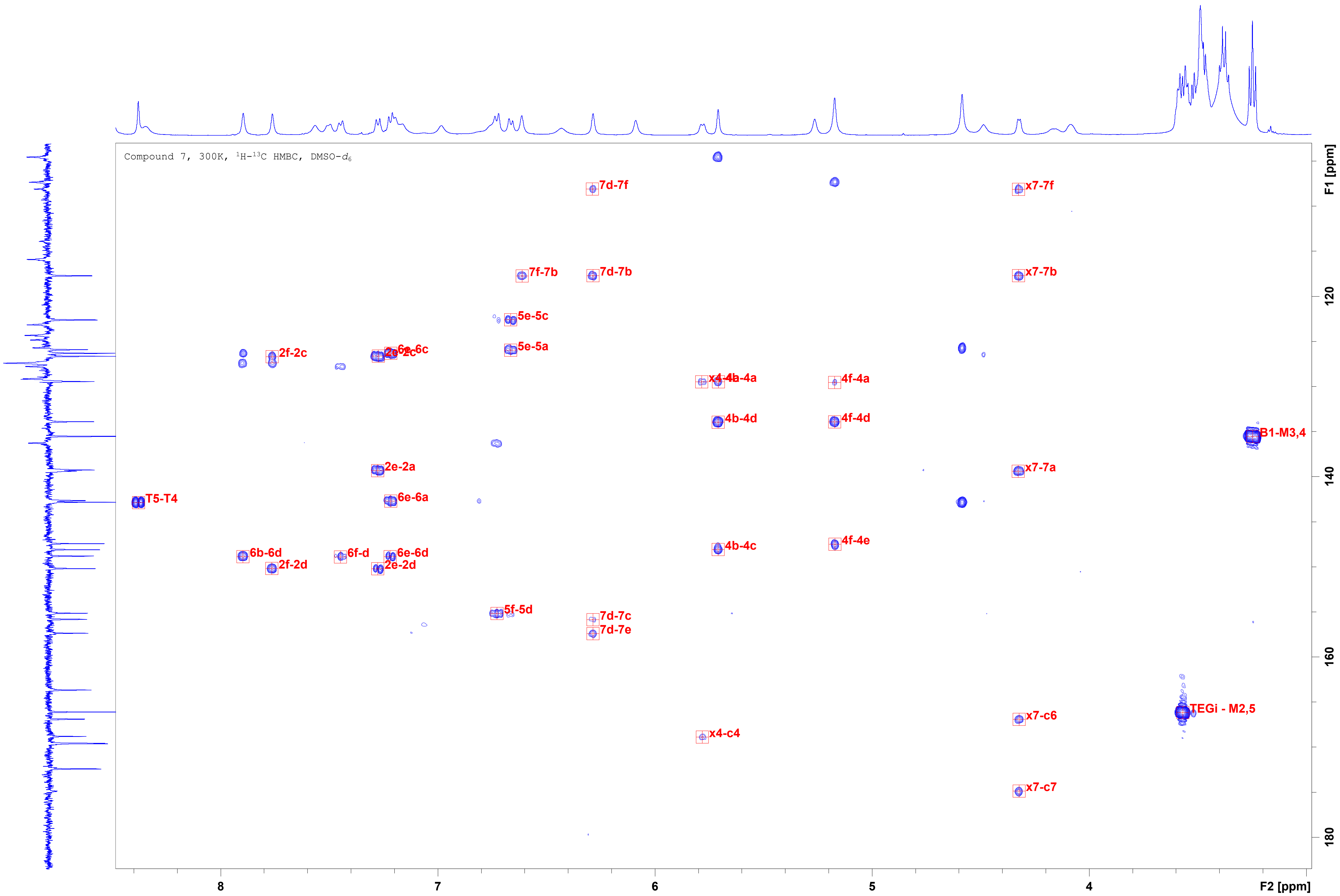

Compound 7, 300K, $^{1}H-^{13}C$ HMBC, DMSO-d_6
7d-7f
x7-7f
7f-7b
7d-7b
x7-7b
5e-5c
2f-2c
5e-5a
4b-4a
4f-4a
4b-4d
4f-4d
B1-M3,4
2e-2a
x7-7a
T5-T4
6e-6a
4b-4c
4f-4e
6b-6d
6f-d
6e-6d
2f-2d
2e-2d
5f-5d
7d-7c
7d-7e
TEGi - M2,5
x7-c6
x4-c4
x7-c7
F1 [ppm]
120
140
160
180
8
7
6
5
4
F2 [ppm]

Compound 8

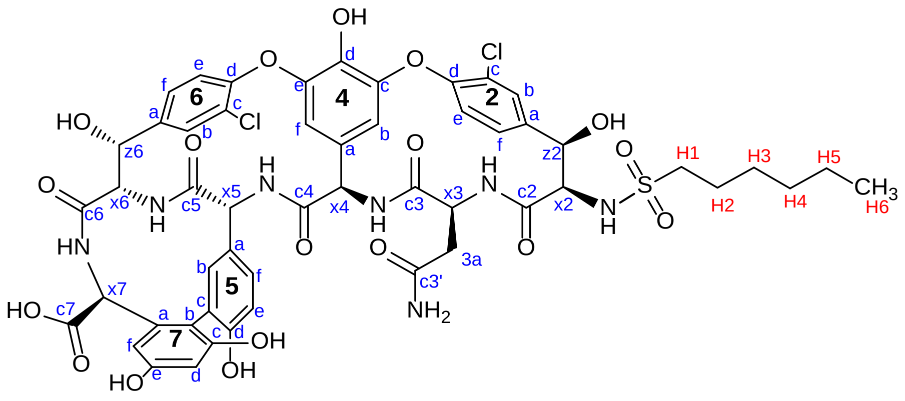

Table S4. NMR data for Compound 8					
Assignment	^{1}H	^{13}C	Assignment	^{1}H	^{13}C
c7	-	175.2	7f	6.61	107.8
c6	-	167.0	4b	5.43	106.4
7e	-	157.0	4f	5.22	104.0
7c	-	156.0	7d	6.33	101.4
4e	-	147.8	z6	5.16	70.8
2d	-	150.5	z2	5.19	70.9
6d	-	148.9	x6	4.20	63.0
2a	-	139.1	x2	4.18	61.9
7a	-	139.2	x7	4.30	59.2
4d	-	134.5	x4	5.63	54.3
2b	7.16	128.5	x5	4.43	53.5
5a	-	125.6	x3	4.61	50.5
6b	7.85	127.8			
5f	6.71	124.1	H1	3.06	52.5
6f	7.46	127.2	H2	1.68	23.3
2f	7.61	128.2	H3	1.36	26.9
(2e)	7.23	123.2	H4	1.24	29.1
(6e)	7.25	124.4	H5	1.26	21.6
5c	-	122.8	H6	0.86	13.5
7b	-	117.7			
5e	6.71	115.9			

( ) = ambiguous

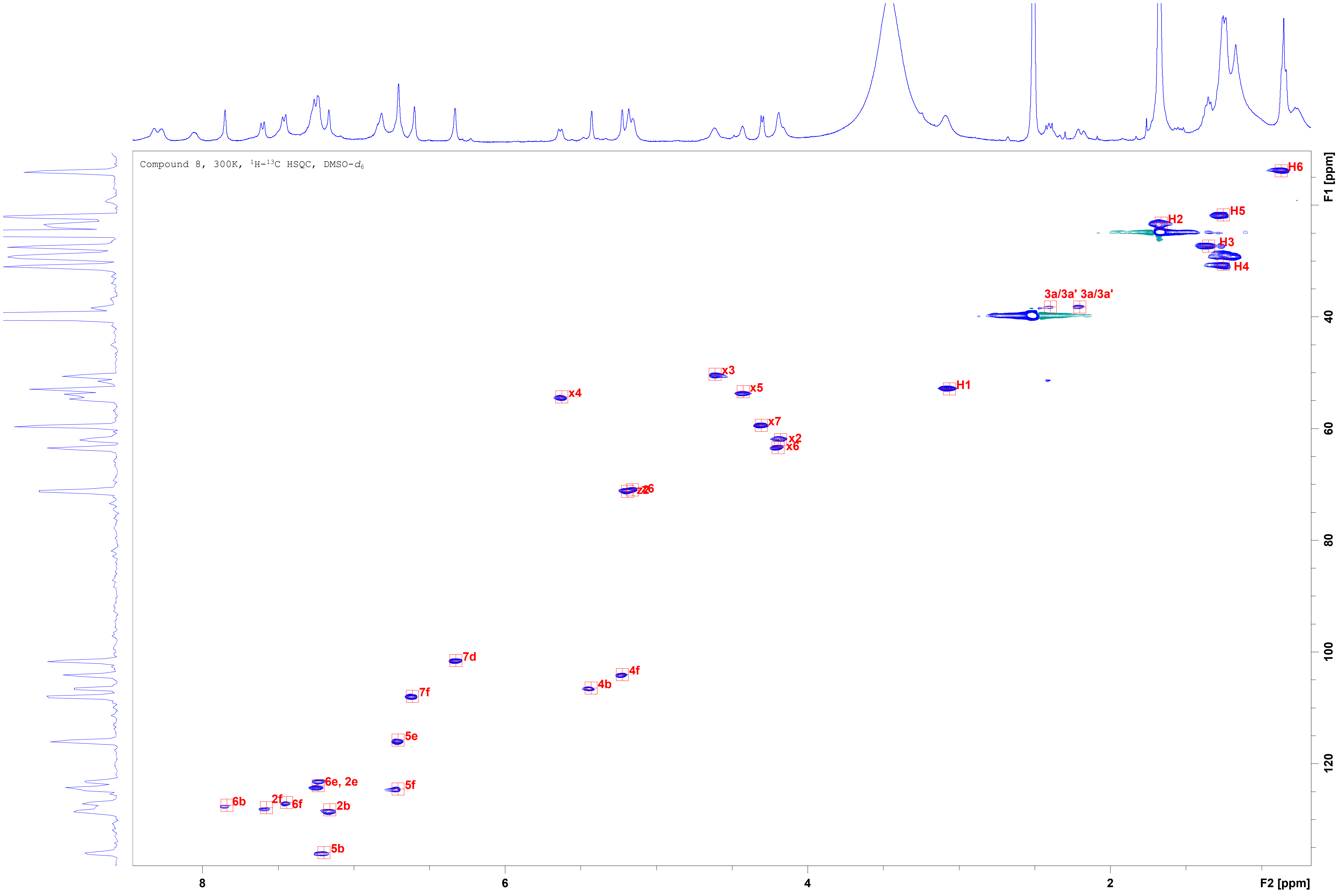

Compound 8, 300K, 1H-13C HSQC, DMSO-d6
H6
H5
H2
H3
H4
3a/3a' 3a/3a'
x3
x4
x5
H1
x7
x2
x6
z2
z6
7d
4f
4b
7f
5e
5f
6e, 2e
2f
6b
6f
2b
5b
F1 [ppm]
40
60
80
100
120
8
6
4
2
F2 [ppm]

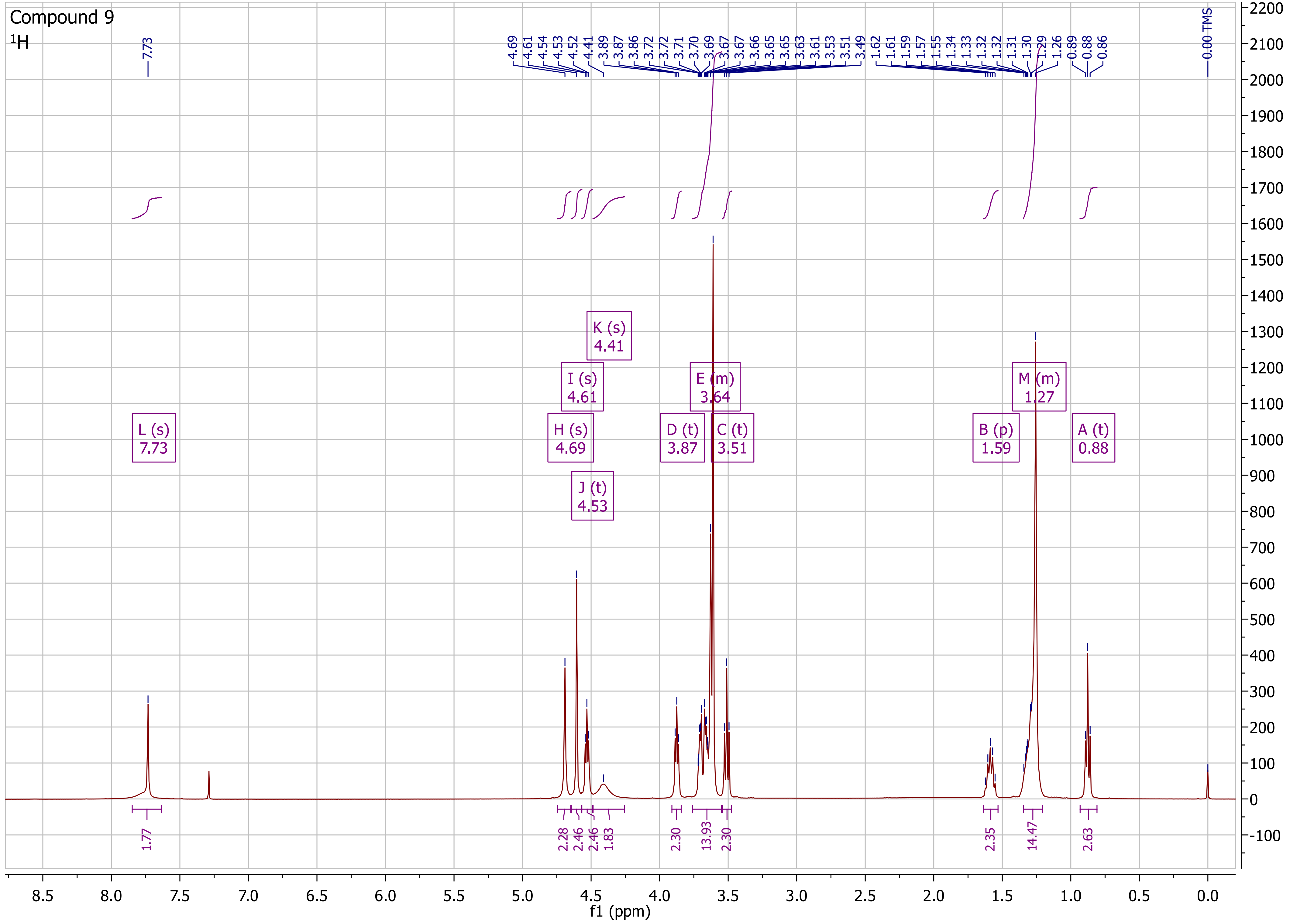

Compound 9
^{1}H
7.73
4.69 4.61 4.54 4.53 4.52 4.41 3.89 3.87 3.86 3.72 3.72 3.71 3.70 3.69 3.67 3.67 3.66 3.65 3.65 3.63 3.61 3.53 3.51 3.49 1.62 1.61 1.59 1.57 1.55 1.34 1.33 1.32 1.32 1.31 1.30 1.29 1.26 0.89 0.88 0.86
0.00 TMS
K (s) 4.41
I (s) 4.61
E (m) 3.64
M (m) 1.27
L (s) 7.73
H (s) 4.69
D (t) 3.87
C (t) 3.51
B (p) 1.59
A (t) 0.88
J (t) 4.53
1.77 2.28 2.46 2.46 1.83 2.30 13.93 2.30 2.35 14.47 2.63
f1 (ppm)

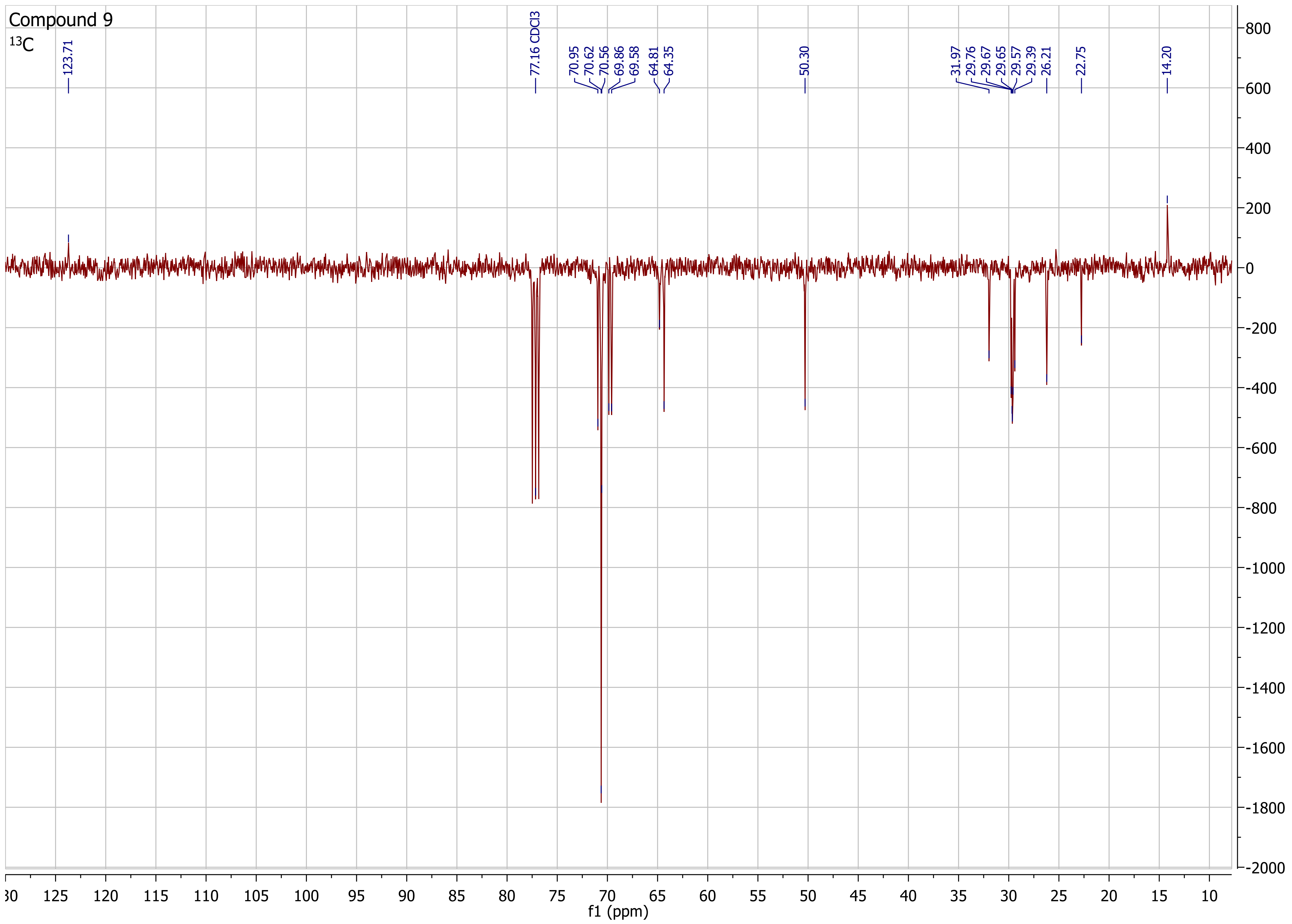

Compound 9
^{13}C
123.71
77.16 CDCl3
70.95
70.62
70.56
69.86
69.58
64.81
64.35
50.30
31.97
29.76
29.67
29.65
29.57
29.39
26.21
22.75
14.20
f1 (ppm)

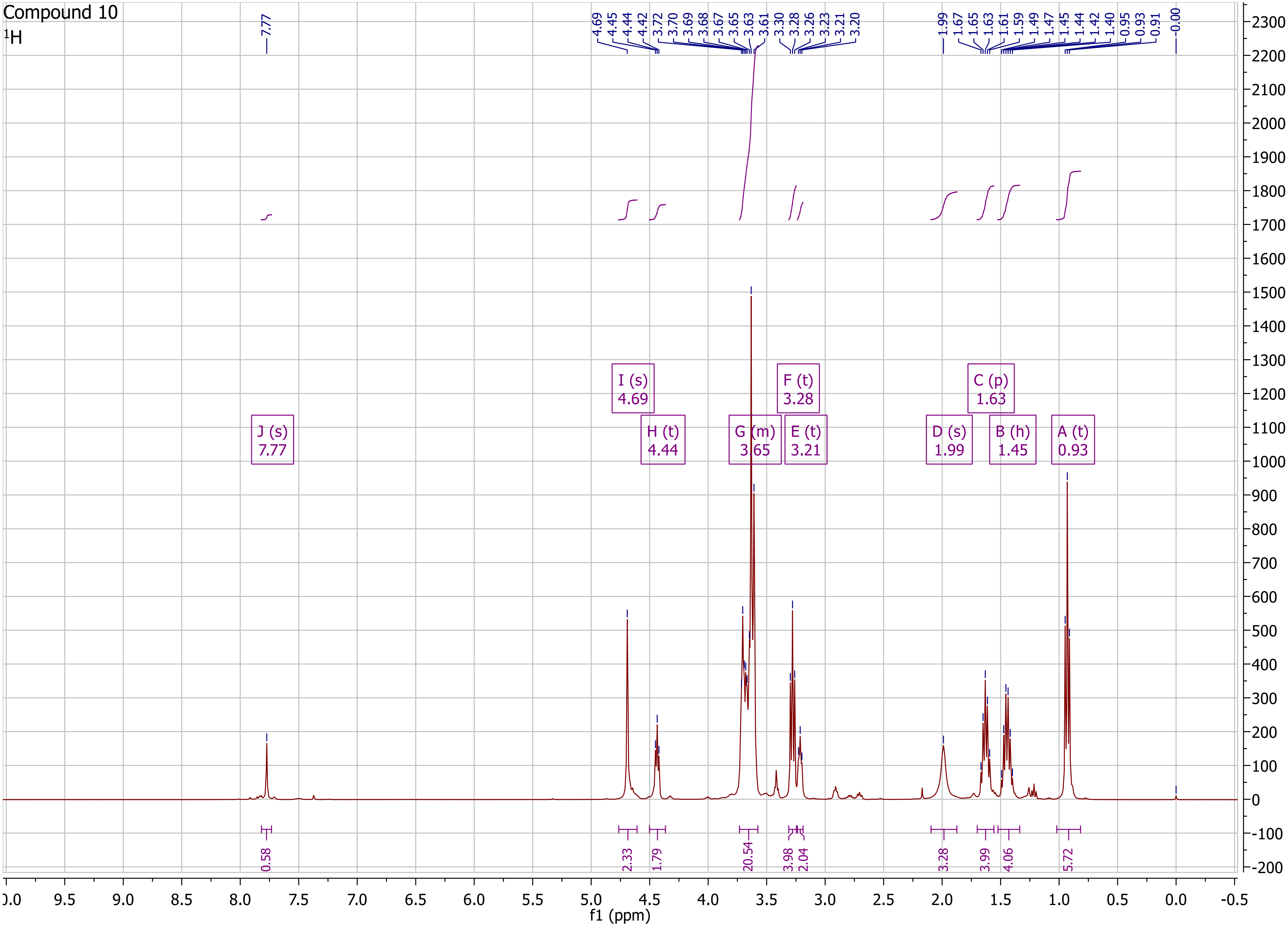
Compound 10
1H
7.77
4.69
4.45
4.44
4.42
3.72
3.70
3.69
3.68
3.67
3.65
3.63
3.61
3.30
3.28
3.26
3.23
3.21
3.20
1.99
1.67
1.65
1.63
1.61
1.59
1.49
1.47
1.45
1.44
1.42
1.40
0.95
0.93
0.91
-0.00
J (s) 7.77
I (s) 4.69
H (t) 4.44
G (m) 3.65
F (t) 3.28
E (t) 3.21
D (s) 1.99
C (p) 1.63
B (h) 1.45
A (t) 0.93
0.58
2.33
1.79
20.54
3.98
2.04
3.28
3.99
4.06
5.72
f1 (ppm)

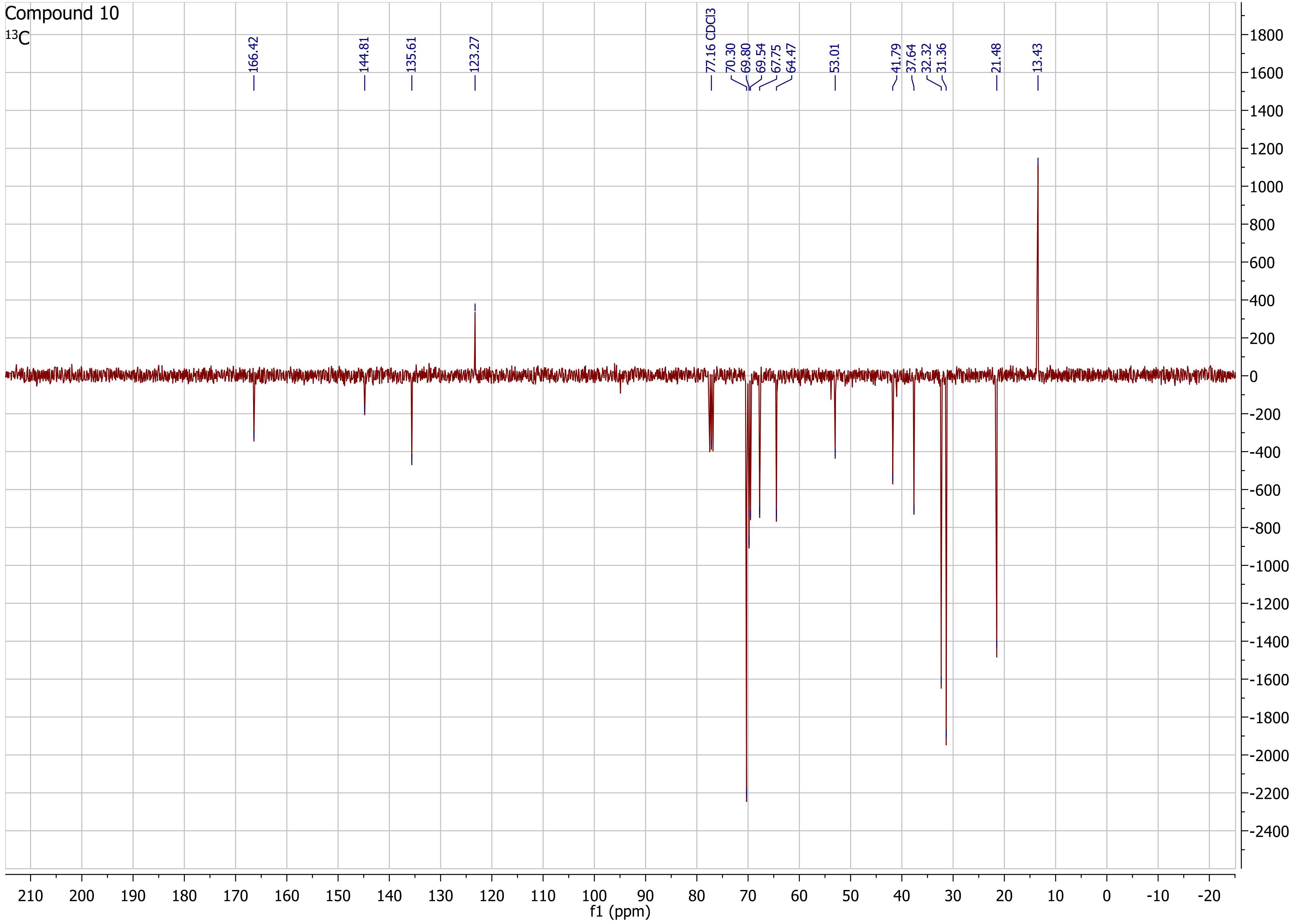
Compound 10
^{13}C
166.42
144.81
135.61
123.27
77.16 CDCl3
70.30
69.80
69.54
67.75
64.47
53.01
41.79
37.64
32.32
31.36
21.48
13.43
f1 (ppm)

Compound 11

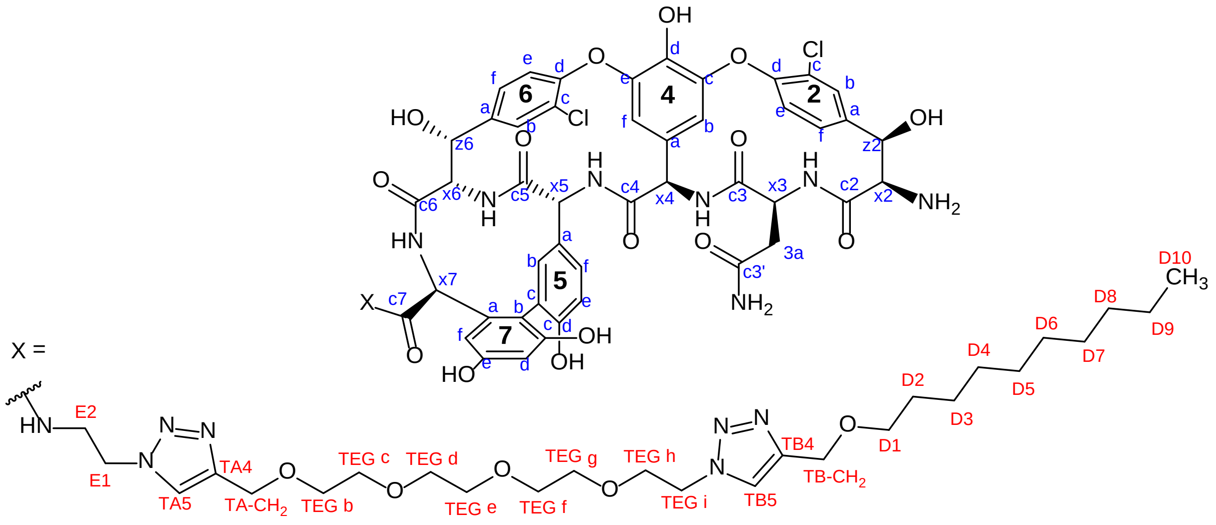

Table S5. NMR data for Compound 11								
Assignment	^{1}H	^{13}C	Assignment	^{1}H	^{13}C	Assignment	^{1}H	^{13}C
c3'(c3)	-	170.2	6f	7,47	126,7	TA-CH_2	4.53	63.0
c7	-	170.5	6c	-	125,7	TA4	-	143.7
c5	-	169.1	2f	7,59	127,6	TA5	8.03	124.1
c6	-	168.3	2e	7,20	123,1	TB-CH_2	4.47	63.0
c2	-	170.1	5c	-	121,8	TB4	-	144.1
7e	-	156.9	7b	-	117,9	TB5	8.04	123.9
7c	-	156.3	5e	6,73	116,2	TEG-CH_2	3.49	69.4
5d	-	155.0	7f	6,20	105,9	TEG-CH_2	3.52	69.4
(4e)	-	147.3	4b	5,48	106,3	TEG b	3.56	68.6
(4c)	-	147.1	4f	5,27	104,0	TEG h	3.82	68.4
2d	-	150.2	7d	6,39	101,9	TEG i	4.52	48.9
6d	-	149.0				D1	3.42	69.2
6a	-	141.6	z6	5,28	71,1	D2	1.50	28.9
2a	-	138.8	z2	5,10	71,5	D3	1.27	25.4
7a	-	137.1	x6	4,25	61,7	D4	1.26-1.23	28.7
5b	7.25	135.3	x2	3,62	60,9	D5		28.9
4d	-	133.7	x7	4,39	57,5	D6		29.0
4a	-	128.7	x4	5,70	53,9	D7		29.1
2c	-	126.5	x5	4,45	53,5	D8	1.24	31.1
2b	7.17	128.6	x3	4,70	50,6	D9	1.27	21.8
5a	-	126.2				D10	0.86	13.8
6b	7.84	127.2	3a	2,30;2,17	38,6	E1	3.61	39.0
5f	6.79	125.1				E2	4.53	48.2

( ) = ambiguous

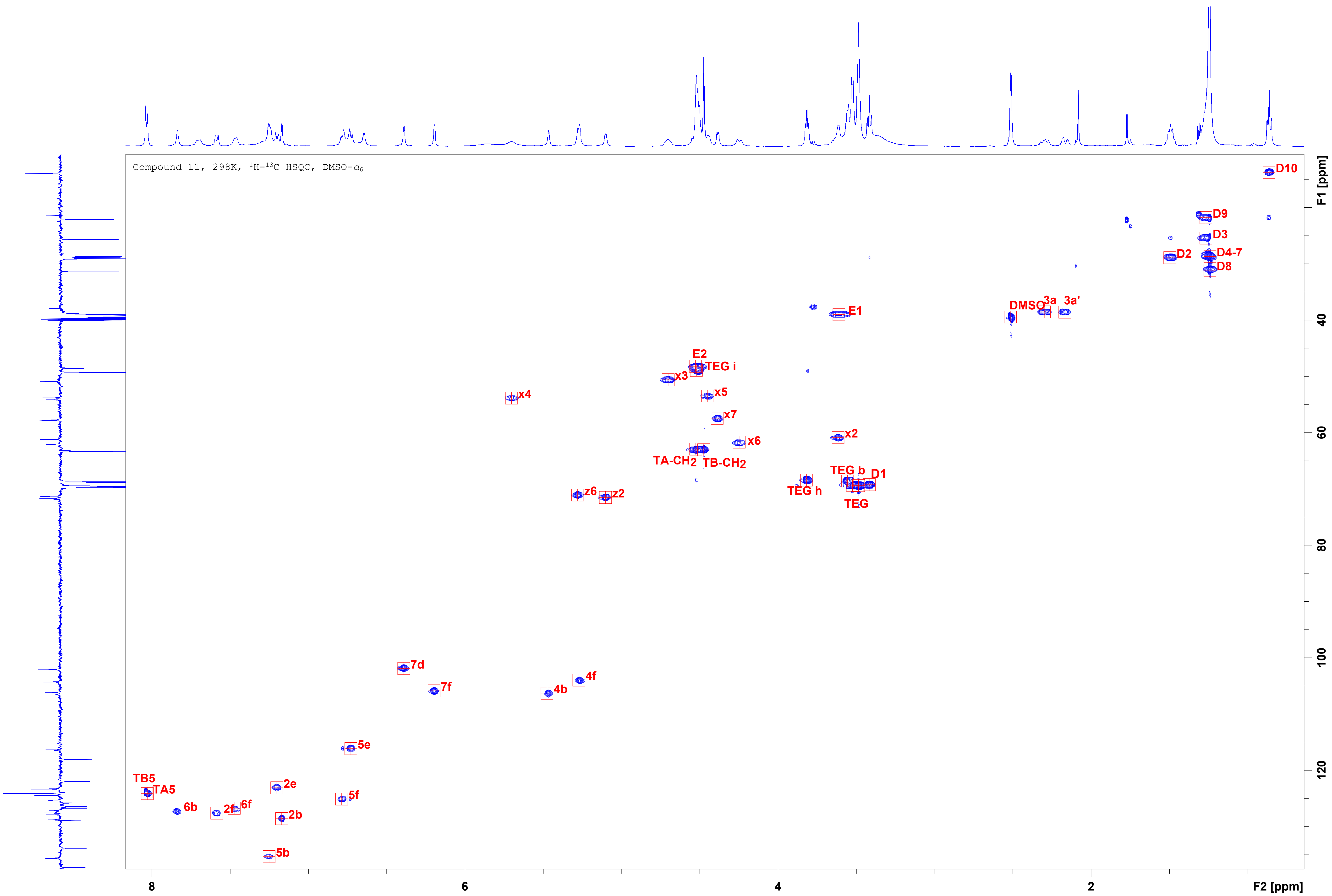
Compound 11, 298K, ¹H-¹³C HSQC, DMSO-d6
D10
D9
D3
D2
D4-7
D8
DMSO
3a
3a'
E1
E2
TEG i
x3
x4
x5
x7
x2
x6
TA-CH2
TB-CH2
TEG b
D1
TEG h
TEG
z6
z2
7d
7f
4b
4f
5e
TB5
TA5
2e
5f
6b
2f
6f
2b
5b
F1 [ppm]
F2 [ppm]

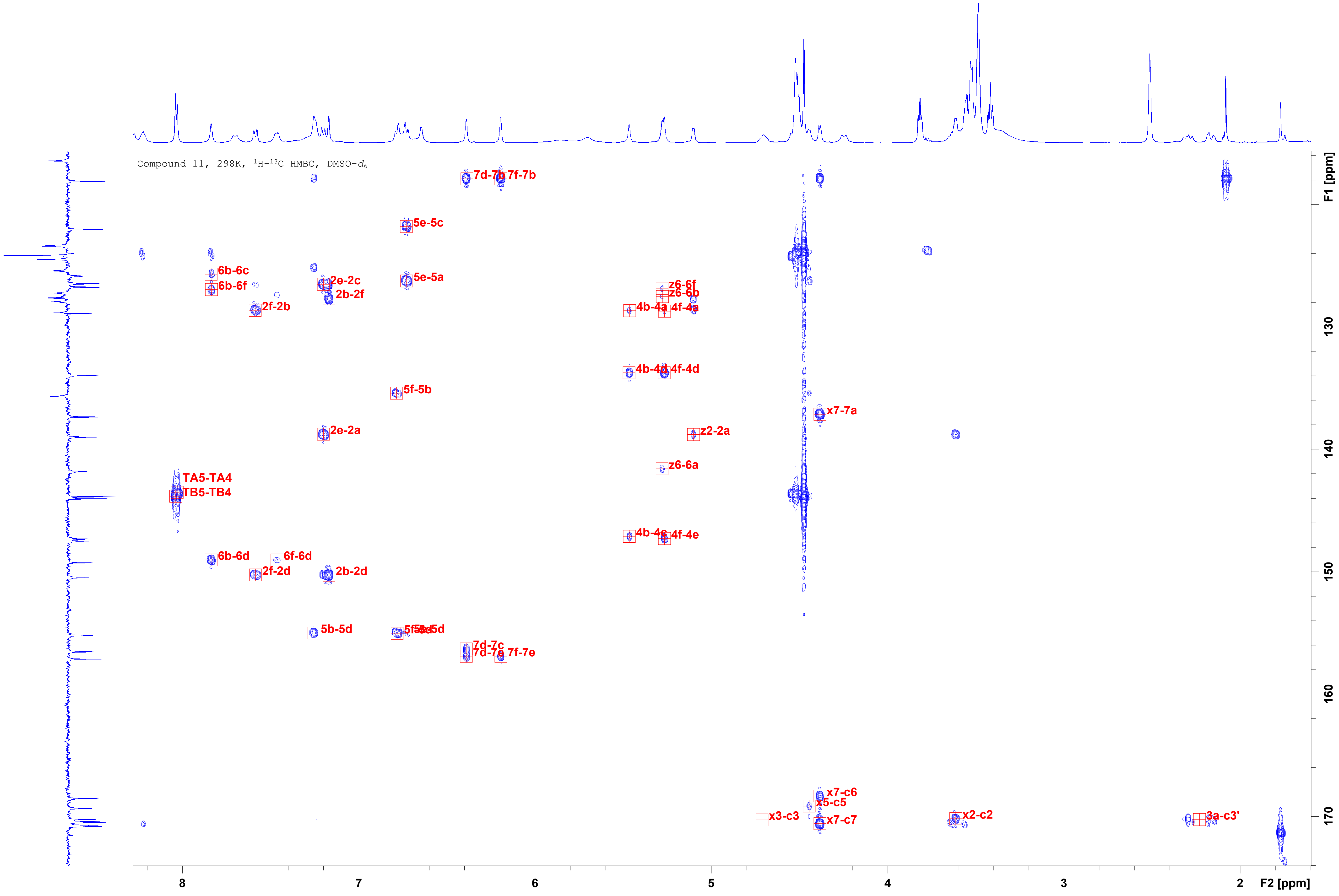
Compound 11, 298K, ^1H-^{13}C HMBC, DMSO-d_6
7d-7b
7f-7b
5e-5c
6b-6c
2e-2c
5e-5a
6b-6f
2b-2f
z6-6f
z6-6b
2f-2b
4b-4a
4f-4a
4b-4d
4f-4d
5f-5b
x7-7a
2e-2a
z2-2a
z6-6a
TA5-TA4
TB5-TB4
4b-4c
4f-4e
6b-6d
6f-6d
2f-2d
2b-2d
5b-5d
7d-7c
7d-7e
7f-7e
x7-c6
x5-c5
x3-c3
x7-c7
x2-c2
3a-c3'
F1 [ppm]
F2 [ppm]
130
140
150
160
170
8
7
6
5
4
3
2

Compound 12

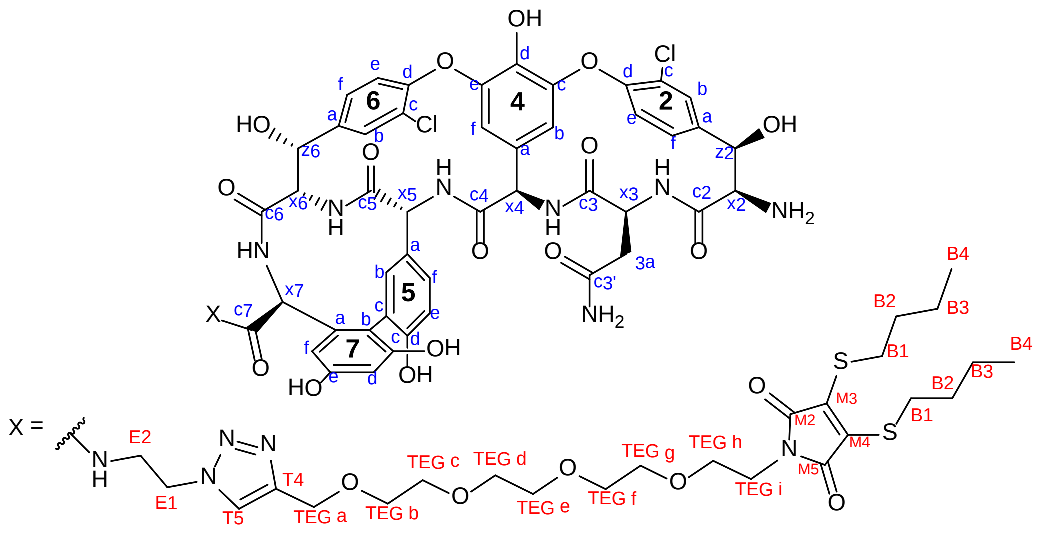

Table S6. NMR data for Compound 12					
Assignment	^{1}H	^{13}C	Assignment	^{1}H	^{13}C
c3	-	170.5	4f	5.26	104.0
c7	-	170.7	7d	6.38	101.7
c5	-	169.2	z6	5.27	71.1
c6	-	168.4	z2	5.09	71.4
c2	-	170.3	x6	4.24	61.8
c3'	-	170.5	x2	3.61	60.9
7e	-	157.0	x7	4.38	57.6
7c	-	156.4	x4	5.68	53.9
5d	-	155.0	x5	4.44	53.5
(4e)	-	147.4	x3	4.69	50.6
(4c)	-	147.3	3a,a’	2.28;2.16	38.4
2d	-	150.4			
6d	-	149.2	T4	-	143.8
2a	-	138.9	T5	8.02	124.3
7a	-	137.3			
5b	7.25	135.3	M2,5	-	166.0
4d	-	133.9	M3,4	-	135.5
2b	7.17	128.6			
5a	-	126.5	B1	3.26	30.3
6b	7.84	127.5	B2	1.56	31.7
5f	6.78	125.2	B3	1.38	20.7
6f	7.47	127.0	B4	0.87	13.1
6c	-	125.9	E1	3.60	38.8
2f	7.58	127.7	E2	4.51	47.9
2e	7.19	123.1	TEG-CH_2	3.55	68.8
6e	7.29	123.1	TEG-CH_2	3.51	69.3
5c	-	121.9	TEG-CH_2	3.47	69.4
7b	-	118.0	TEG a	4.52	63.1
5e	6.72	116.1	TEG h	3.52	66.5
7f	6.19	106.0	TEG i	3.57	37.4
4b	5.46	106.4			

( ) = ambiguous

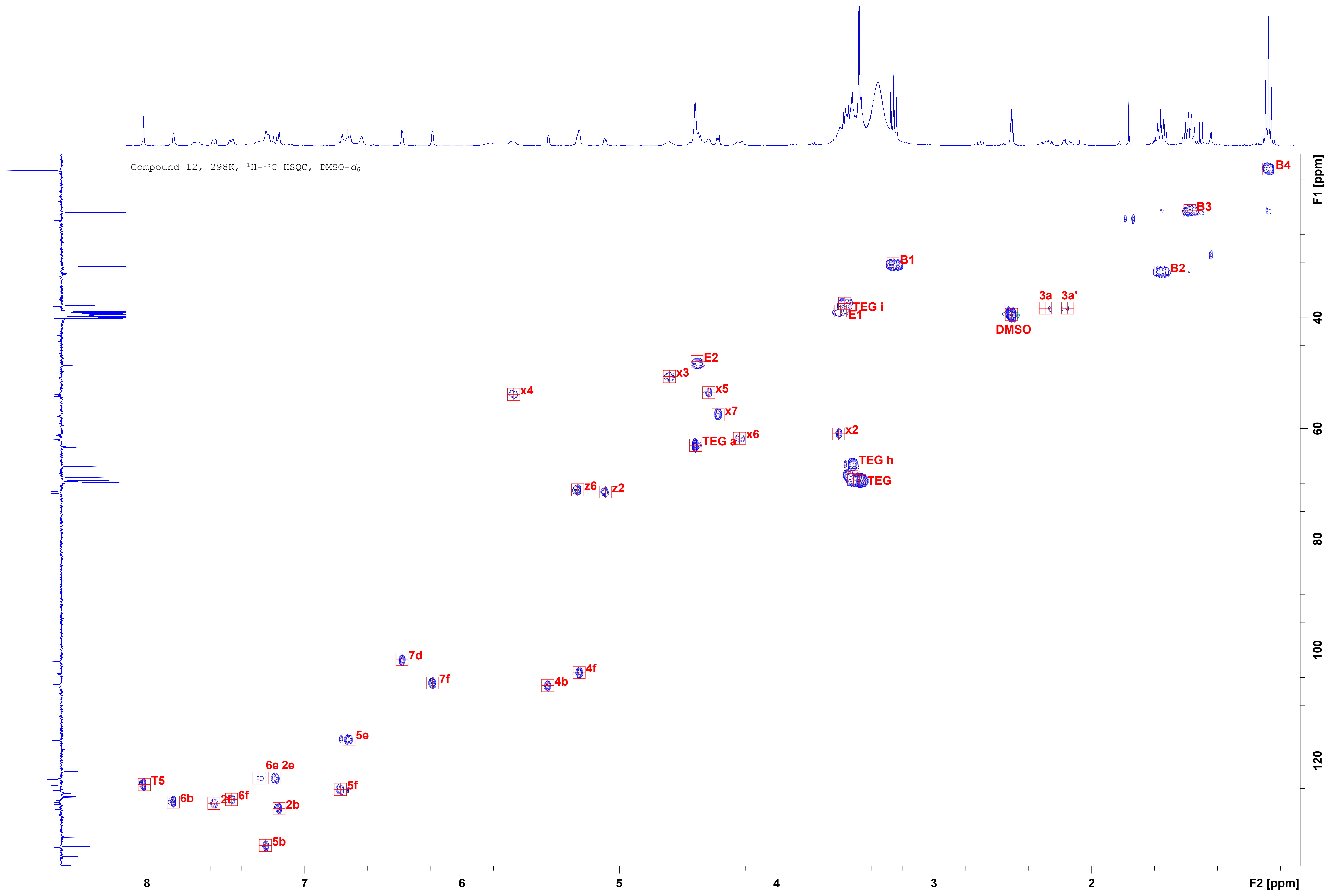
Compound 12, 298K, 1H-13C HSQC, DMSO-d6
B4
B3
B1
B2
3a
3a'
TEG i
E1
DMSO
E2
x3
x4
x5
x7
x2
x6
TEG a
TEG h
TEG
z6
z2
7d
4f
7f
4b
5e
6e 2e
T5
5f
6b
2f
6f
2b
5b
F1 [ppm]
F2 [ppm]

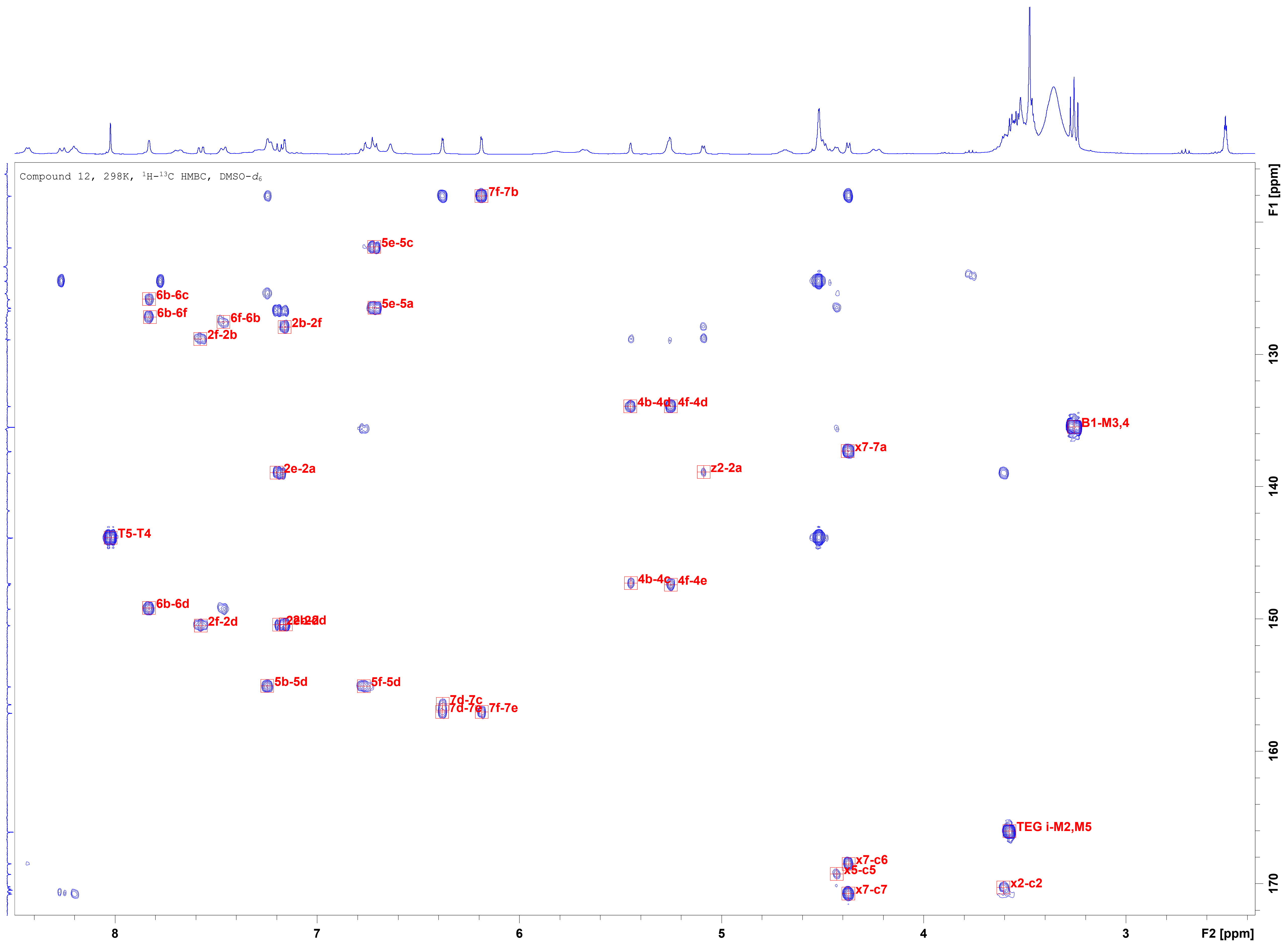

Compound 12, 298K, $^{1}H-^{13}C$ HMBC, DMSO-d_6
7f-7b
5e-5c
6b-6c
6b-6f
6f-6b
2b-2f
2f-2b
5e-5a
4b-4d
4f-4d
B1-M3,4
x7-7a
2e-2a
z2-2a
T5-T4
4b-4c
4f-4e
6b-6d
2f-2d
5b-5d
5f-5d
7d-7c
7d-7e
7f-7e
TEG i-M2,M5
x7-c6
x5-c5
x7-c7
x2-c2
F1 [ppm]
130
140
150
160
170
8
7
6
5
4
3
F2 [ppm]

Compound 13

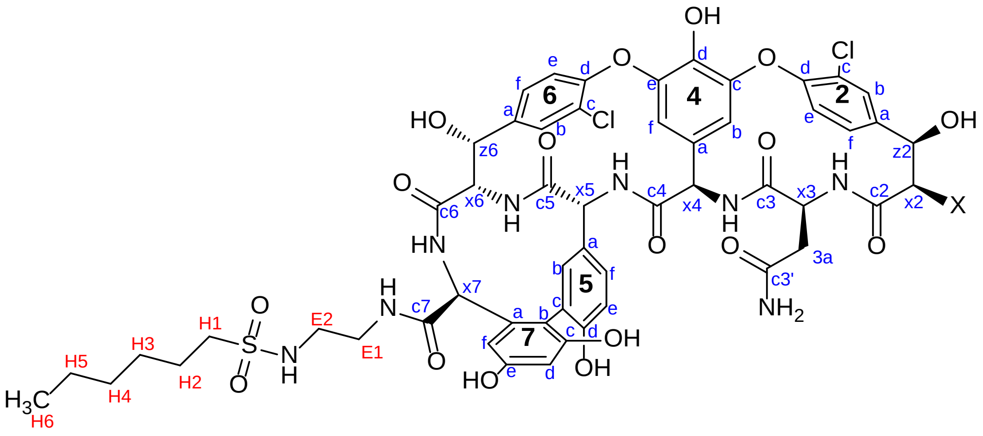

Table S7. NMR data for Compound 13

Assignment	^{1}H	^{13}C	Assignment	^{1}H	^{13}C	Assignment	^{1}H	^{13}C
c3	-	170.3	6b	7.84	127.4	x6	4.23	61.9
c7	-	170.4	5f	6.78	125.8	x2	3.61	61.6
c5	-	169.2	6f	7.43	127.0	x7	4.37	57.5
c2	-	170.3	6c	-	125.7	x4	5.70	54.0
7e	-	157.0	2f	7.57	127.6	x5	4.43	53.6
7c	-	156.3	(2e)	7.19	123.1	x3	4.69	50.6
5d	-	155.0	(6e)	7.11	122.2	3a,a’	2.29;2.18	38.5
(4e)	-	147.4	5c	-	121.8			
(4c)	-	147.2	7b	-	118.0	E1	3.25	39.0
6d	-	149.1	5e	6.74	116.1	E2	3.05	41.3
2a	-	138.9	7f	6.22	106.0			
7a	-	137.4	4b	5.47	106.5	H1	3.01	50.9
5b	7.25	135.4	4f	5.27	104.1	H2	1.64	22.9
4d	-	133.9	7d	6.39	101.9	H3	1.38	27.0
4a	-	128.8				H4	1.27	30.6
2b	7.17	128.6	z2	5.10	71.6	H5	1.28	21.5
5a	-	126.4	z6	5.27	71.1	H6	0.87	13.4

( ) = ambiguous

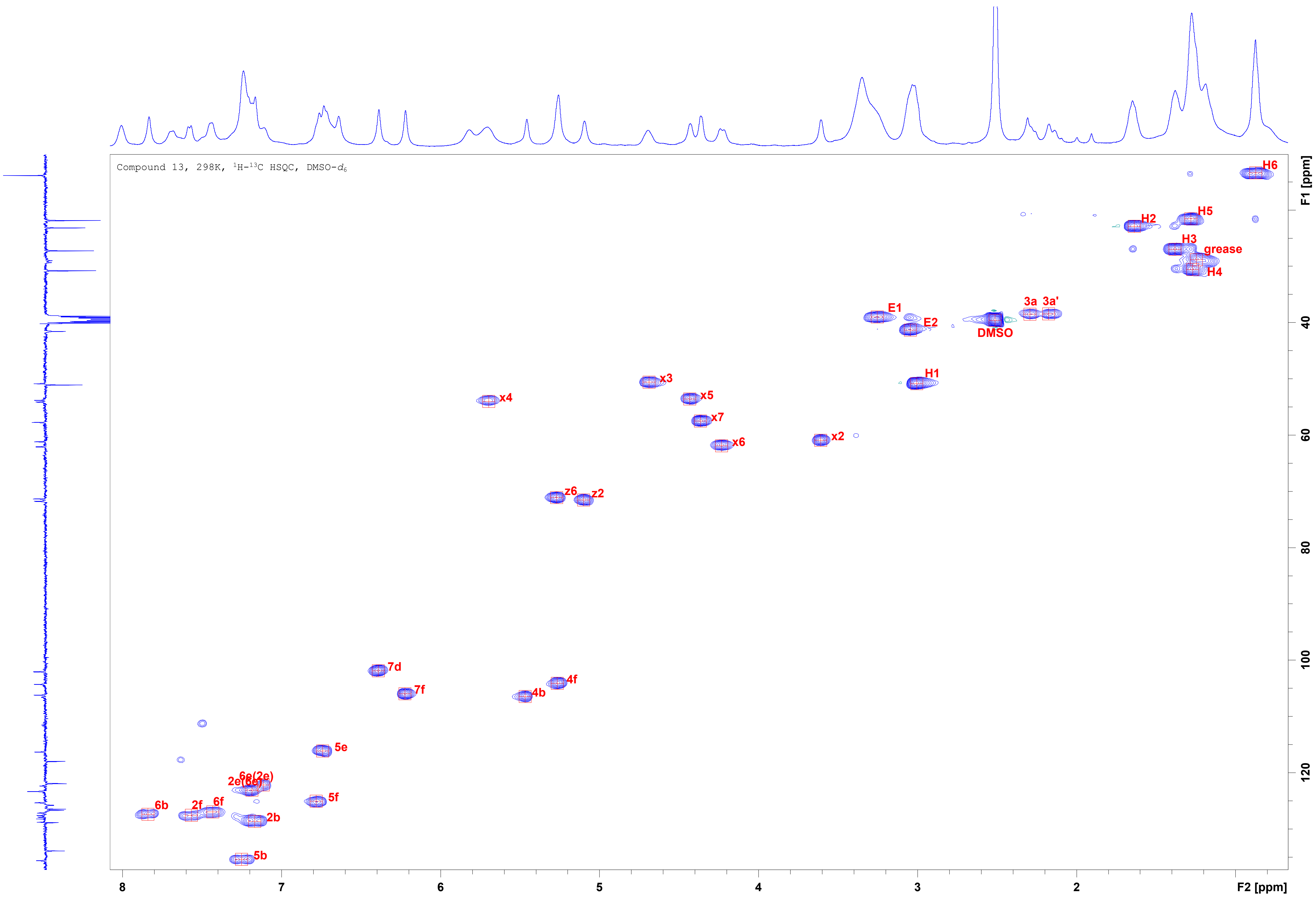

Compound 13, 298K, ¹H-¹³C HSQC, DMSO-d6
H6
H2
H5
H3
grease
H4
3a
3a'
E1
E2
DMSO
H1
x3
x4
x5
x7
x2
x6
z6
z2
7d
7f
4f
4b
5e
6e(2e)
2e(6e)
6b
2f
6f
2b
5f
5b
F1 [ppm]
F2 [ppm]

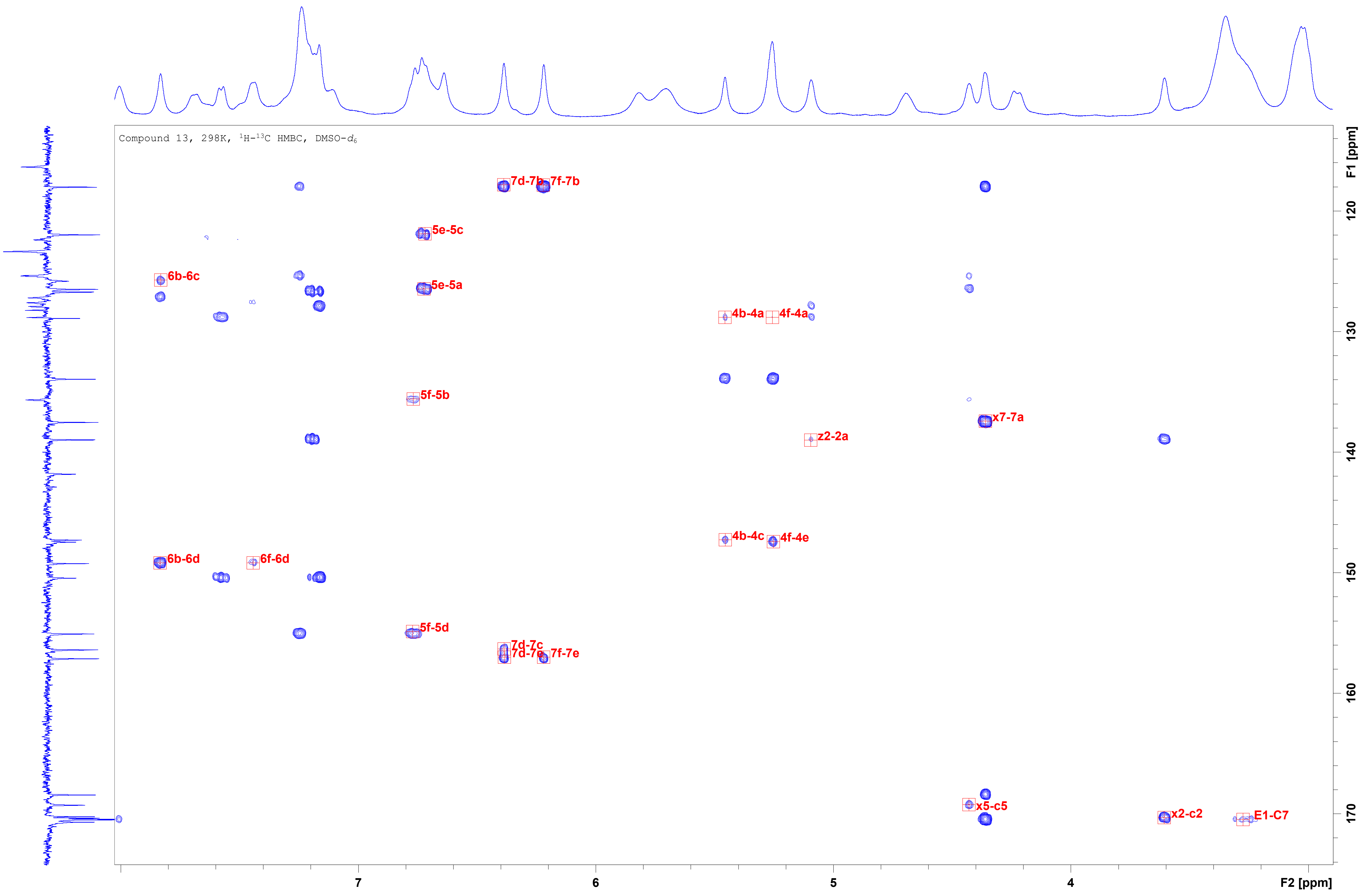

Compound 13, 298K, ^{1}H-^{13}C HMBC, DMSO-d_6
7d-7b
7f-7b
5e-5c
6b-6c
5e-5a
4b-4a
4f-4a
5f-5b
x7-7a
z2-2a
4b-4c
4f-4e
6b-6d
6f-6d
5f-5d
7d-7c
7d-7e
7f-7e
x5-c5
x2-c2
E1-C7
F1 [ppm]
F2 [ppm]
120
130
140
150
160
170
7
6
5
4

Table S8. Elemental analysis data (C, H, N, S) for vancomycin derivatives **6-8**, **11-13**.

#	Formula	C		H		N		S	
		Calcd.	Found	Calcd.	Found	Calcd.	Found	Calcd.	Found
**6**	$C_{70}H_{80}Cl_2N_{12}O_{21}$	56.19	56.03	5.39	5.60	11.23	11.10	-	-
**7**	$C_{69}H_{74}Cl_2N_{10}O_{22}S_2$	54.15	53.98	4.87	5.05	9.15	9.05	4.19	4.01
**8**	$C_{52}H_{51}Cl_2N_7O_{18}S$	53.61	53.40	4.41	4.68	8.42	8.22	2.75	2.62
**11**	$C_{72}H_{86}Cl_2N_{14}O_{20}$	56.21	56.04	5.63	5.78	12.75	12.66	-	-
**12**	$C_{71}H_{80}Cl_2N_{12}O_{21}S_2$	54.23	54.03	5.13	5.34	10.69	10.54	4.08	3.97
**13**	$C_{54}H_{57}Cl_2N_9O_{17}S$	53.73	53.58	4.76	4.96	10.44	10.26	2.66	2.54
